# High affinity mAb infusion can enhance maximum affinity maturation during HIV Env immunization

**DOI:** 10.1016/j.isci.2024.109495

**Published:** 2024-03-11

**Authors:** Peter Thomas, Chloe Rees-Spear, Sarah Griffith, Luke Muir, Emma Touizer, Raiees Andrabi, Richard Priest, Jennifer Percival-Alwyn, Darryl Hayward, Amanda Buxton, William Traylen, Benny Chain, Trevor Wattam, Irene Sanjuan Nandin, Laura E. McCoy

**Affiliations:** 1 Institute of Immunity and Transplantation, Division of Infection and Immunity, University College London, London WC1E 6BT, UK; 2 Biopharmaceutical Molecular Discovery Group, GSK, Gunnels Wood Rd, Stevenage SG1 2NY, UK; 3 Department of Medicine, University of Pennsylvania, Philadelphia 19104, PA, USA; 4 In vivo Sciences and Delivery, GSK, Park Road, Ware SG12 0DP, UK; 5 Immunology Research Unit, GSK, Gunnels Wood Rd, Stevenage SG1 2NY, UK; 6 Department of Computer Science, University College London, London WC1E 6EA, UK

**Keywords:** Immunology, Immune response, Virology

## Abstract

Antigen-specific antibody infusion is known to enhance or suppress germinal center (GC) responses depending on the affinity of the infusion. We hypothesized that infusing monoclonal antibodies (mAbs) of escalating affinity during an immunization regimen may progressively escalate selection pressure on competing B cells, increasing their affinity. To test this, we immunized mice with HIV envelope gp120 and infused CD4 binding-site (CD4bs)-specific mAbs. While mAb infusion reduced somatic hypermutation (SHM) and affinity in most CD4bs-specific B cells, a sub-population was identified with greater SHM and affinity than control. High-throughput sequencing of plasma cells revealed that CD4bs-specific plasma cells possessed elevated SHM after mAb infusion, with phylogenetic tree topology that suggested more rapid differentiation. We therefore conclude, in accordance with other studies, that high-affinity mAb infusion primarily suppresses recruitment of most competing B cells but can increase and expedite affinity maturation of certain epitope-specific B cells.

## Highlights

• CD4bs bnAb infusion in immunization reduced SHM across the bulk B cell population
• MAb infusion in immunization also produced a group of higher affinity CD4bs mAbs
• These higher affinity mAbs used more diverse V_H_ genes than control
• CD4bs mAb infusion raised SHM in certain B cells, expediting plasma cell transition

## Graphical abstract

**Figure:**
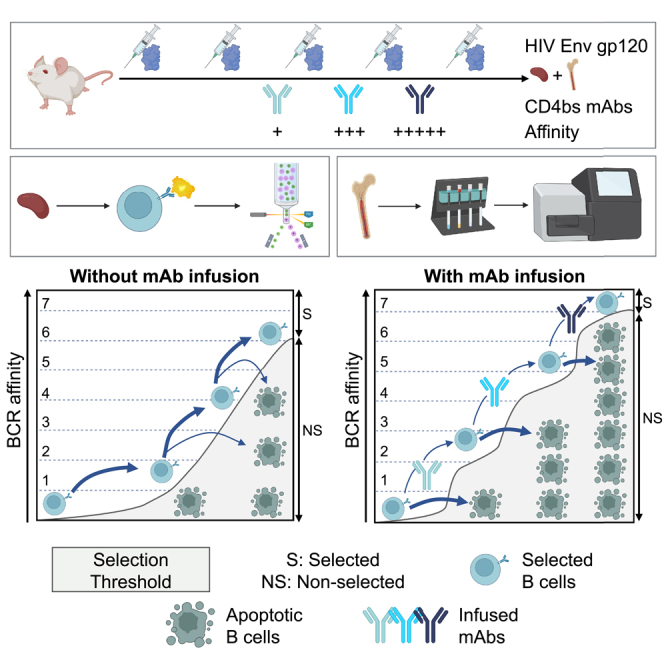

## Introduction

The germinal center (GC) is a highly competitive environment where B cells mature the affinity of their B cell receptors (BCRs) to their cognate antigen. The GC is characterized by repeated rounds of BCR somatic hypermutation (SHM) and rapid cellular division in the dark zone (DZ), followed by affinity-based selection in the light zone (LZ).^1^ In the LZ, B cells next acquire antigen from the follicular dendritic cell (FDC) surface via their BCR.^2^ The amount of antigen captured by each B cell is dictated by the affinity of the BCR for antigen, which correlates with the amount of help the B cell can receive when it later presents the processed antigen to T follicular helper (Tfh) cells, following antigen endocytosis and processing.^3^ Therefore, the Tfh-B cell interaction results in the provision of survival signals to the B cell that trigger re-entry into the DZ to continue BCR maturation or exit from the GC. These post-GC B cells are either memory B cells, which provide a pool of cells for rapid recall in case of re-exposure to the antigen, or long-lived plasma cells that migrate to the bone marrow niches and secrete protective antibody.^4^^,^^5^^,^^6^

Soluble antibody, either produced during the endogenous response or passively transferred, has been demonstrated to modulate the GC.^7^^,^^8^^,^^9^^,^^10^^,^^11^^,^^12^ Notably, soluble antibody can enhance affinity of B cell responses by competing with B cells in the LZ for antigen acquisition, demonstrated by elevating serum affinity toward the nitrophenyl conjugated chicken gamma globulin (NP-CGG) hapten. Indeed, in this system, infusion of a high affinity mAb was able to induce faster acquisition of the higher affinity phenotype than low or intermediate affinity mAbs.^12^ This work would suggest that passive infusion of an epitope-specific mAb during an immunization could increase selection pressure in the GC LZ, only permitting selection of B cells whose BCR affinities exceeded that of the infused mAb. This would produce a pool of BCRs with higher affinity than those possible without the addition of competing mAb. However, other publications have highlighted that high affinity antibodies (either monoclonal or polyclonal) have the ability to repress B cell responses and have an overall negative effect on the affinity output of GC to complex antigens.^8^^,^^13^^,^^14^^,^^15^ Epitope masking by the passive antibody may represent a dominant mechanism behind this.^16^^,^^17^ The role of passive antibody in affinity regulation is therefore complex, with the affinity of the infused mAb being of critical importance in determining suppression vs. enhancement of B cell responses by soluble IgG.^10^^,^^15^^,^^18^

The primary objective of this study was to develop a method for generating higher affinity mAbs in transgenic mice containing human immunoglobulin loci (TIg) mice by reconciling the previously observed issues surrounding passive mAb infusion on the B cell response. We immunized TIg mice with a total of 5 doses of immunogen and infused mAbs of escalating affinity 3 days following boosts 1 to 3. We term this strategy ‘repertoire pruning’, which functions to provide increasingly strong selection pressure during B cell affinity maturation. We hypothesized that this approach could guide the BCR repertoire toward greater affinity rather than repressing the total response.^8^^,^^13^^,^^14^^,^^15^ The gp120 subunit of the MGRM8 strain HIV envelope glycoprotein (Env gp120) was selected as a model immunogen since it elicits antibodies against a range of epitopes that are well characterised.^19^ It has also been found in our lab to have high expression and lack of spontaneous dimerisation following expression. In particular, we assessed the response against the CD4bs epitope, which is recognized by high number of bnAbs predominantly from two germline restricted antibody classes.^20^ Moreover, CD4bs-specific B cells can be isolated via cell sorting more easily using an Env gp120 probe and a counter probe containing the D368R mutation.^21^ This mutation either knocks out or knocks down binding to the CD4bs, and therefore facilitates antigen-specific cell isolation by cell sorting. To discover the effects of mAb infusion on the CD4bs, we isolated CD4bs B cells from spleen and lymph node tissues by single cell sorting and subsequently expressed and characterized the affinity of their mAbs using SPR. We then analyzed the SHM of these CD4bs mAbs and later used their DNA sequences to mine the bone marrow IgG repertoire for putative CD4bs sequences.

## Results

### mAb infusion permits greater affinity maturation toward non-CD4bs epitopes and in a subset of CD4bs-specific B cells

**Figure 1:**
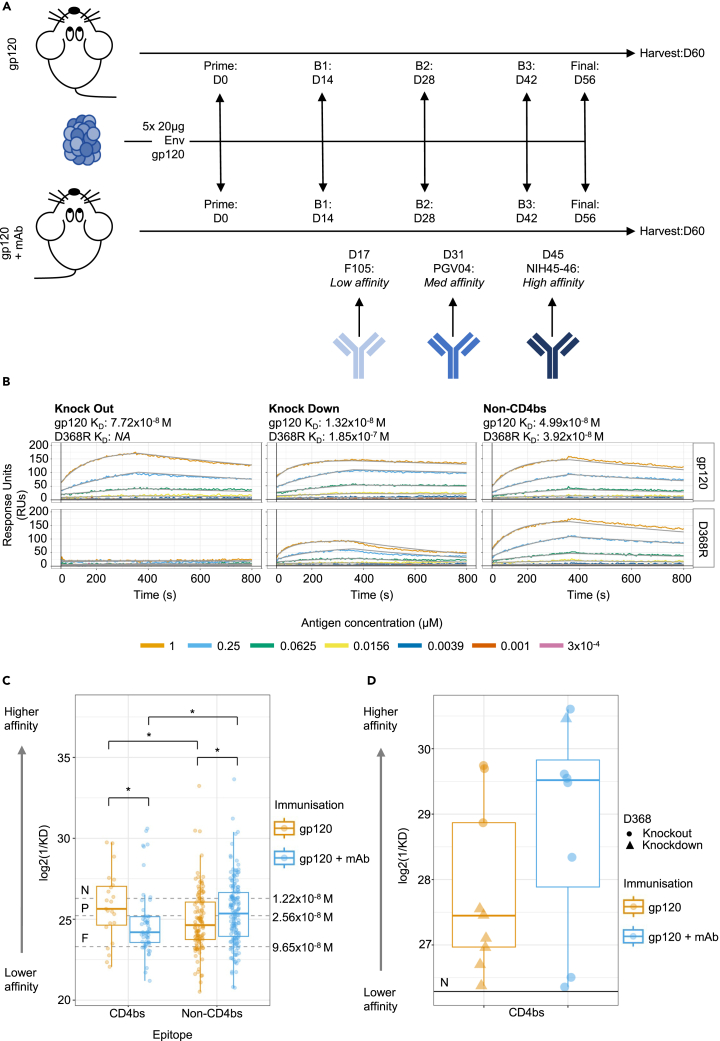
A subset of CD4bs mAbs demonstrate higher affinity following mAb infusion K_D_ analysis of mice from both control and mAb infusion immunization regimens. (A) Immunization schematic depicting dosing of Env gp120 immunogen and CD4bs mAbs used in the repertoire pruning arm. Mice were immunized with 20μg of gp120 on days 0, 14, 28, 42, and 56, with mAb infusion on days 17, 31 and 45, following the second, third and fourth immunization. All immunizations, except the final boost, were delivered to the hock and foreleg lymph nodes (20μg gp120 divided between all sites, 5μg per site). The final boost was delivered intraperitoneally. Serum samples (Test Bleeds; TBs) 1, 2 and 3 were taken to assess the response progression 1 week following the second, third and fourth immunizations, respectively. (B) Illustrative sensorgrams, depicting how CD4bs specificity was assigned. Binding knockouts (sensorgrams 1 & 2) and knock downs (sensorgrams 3 & 4) to the CD4bs are shown, as well as non-CD4bs specific mAbs (sensorgrams 5 & 6). Assignment was based on binding affinity for wild-type Env gp120 and D368R gp120. Since sensorgrams 3 & 4 have a greater than 10-fold lower affinity to the D368R gp120 than to the wild-type, it was assigned as CD4bs specific. (C) Analysis of affinities obtained following SPR. Reciprocal K_D_ values are shown on the y axis after log2 transformation, whereby higher values indicate higher affinity. Unlike B & C, data received additional filtering to comprise only gp120 binding mAbs where the standard deviation of the residuals in the sensorgram was less than or equal to 10. Manual inspection was also applied to verify correct selection, and an additional 22 mAbs whose residual values were outside this range, but sensorgram curve faithfully represented experimental data. Statistical comparison carried out using Mann-Whitney tests (∗∗∗p ≤ 0.001, ∗∗p ≤ 0.01, ∗p < 0.05). The N, P, and F annotations reflect the affinities of NIH45-46, PGV04, and F105 mAbs respectively. (D) Analysis of only CD4bs specific B-cells with a K_D_ value greater than NIH45-46 (the highest affinity mAb infused). Shape of the points reflects if the binding of the mAb was wholly knocked out by the D368R mutation. Statistical analysis carried out with Mann-Whitney test (p = 0.3704). All boxplots illustrate the distribution of quantiles 1 and 3 (Q1 and Q3), with the horizontal line representing the median. Whiskers represent Q3 or Q1 +/- the interquantile range.

To establish the effects of mAb infusion on the B cell response, two groups of 10 TIg mice were immunized with 5 × 20μg doses of Env gp120 spaced two weeks apart, with 1 group receiving repeated mAb infusion throughout the regimen (Figure 1A). In the mAb infusion group, 45μg of the selected CD4bs-specific mAb was injected into the tail vein 3 days following immunizations 2, 3, and 4. The selected CD4bs bnAbs were F105, PGV04 and NIH45-46, since they all target the CD4bs with escalating affinity. These affinities were 9.6x10^−8^M, 2.56x10^−8^M and 1.22x10^−8^M, respectively (indicated on the graph in Figure 1C). Prior to infusion, these mAbs were expressed in a hybrid murine IgG1 format (muFC mAb), and it was confirmed that these mAbs retained equivalent recognition of the CD4bs. This immunization strategy follows standard practice for therapeutic mAb generation, with the interval between immunization and mAb infusion following previous work in this field.^12^

CD4bs and whole Env-gp120 specific IgG serum titers were assessed 7 days following immunizations 2, 3, and 4, and upon tissue harvest. These demonstrated statistically similar responses to each antigen within each serum sample, highlighting that the mAb infusion did not restrain serum IgG responses (Figure S1). Upon tissue harvest, spleen and lymph nodes were pooled from mice within each immunization group, and B cells recognizing the CD4bs were isolated by single cell sorting. Cells selected were live B220+/IgM-/IgG+ single lymphocytes, binding wild-type MGRM8 Env gp120 (CRF Clade AG), but not BG505 D368R Env gp120 (Clade A) (Figures S2A and S2B). The counter probe used the BG505 viral strain to enrich for CD4bs-specific cells while also allowing selection of some non-CD4bs cells, to analyze any non-epitope specific effects of the mAbs.

Isolation of antigen-specific cells from this general B cell population (B220+/IgG+) represents standard practice within therapeutic antibody discovery. Non-IgG, non-IgM B cells were considered to be either IgA expressing or cells which have down-regulated surface Ig. These cells were deposited into individual wells of a 96 well plate containing lysis buffer, and V_H_V_K_ mRNA was recovered and amplified by RT-PCR. The mAb encoding DNA was then transfected and expressed using HEK293F cells, before the supernatants were harvested and tested by SPR to experimentally verify mAb specificity and affinity. MAbs which showed either complete loss of binding or diminished binding (10-fold reduction in K_D_) to the D368R mutant antigen were classified as CD4bs mAbs (Figure 1B). Unlike spleen and lymph node tissues, bone marrow tissues from these mice were retained and processed separately for bulk IgG sequencing after enrichment for plasma cells.

To assess if mAb infusion altered B cell targeting of the CD4bs, a χ^2^ test was carried out to compare the frequencies of B cells targeting each epitope group, which demonstrated no significant alteration in Env gp120 targeting following mAb infusion (p = 0.199). Having discovered the epitope specificity of the mAbs isolated from each immunization regimen and that there was no obvious difference in their targeting, their K_D_ values were able to be compared. Firstly, considering the CD4bs mAbs, the average K_D_ following mAb infusion was significantly lower than in the control group (Figure 1C; p = 0.019), which reflects that access to the CD4bs has been restricted by mAb infusion. Control immunized mice also displayed significantly higher average affinity toward the CD4bs compared to non-CD4bs epitopes (p = 0.046), while the opposite was true for mice receiving mAb infusion during the immunization (p = 0.004). Furthermore, affinity to the non-CD4bs epitopes was significantly higher in mice receiving mAb infusion than control mice receiving only Env gp120 (p = 0.018). This demonstrates that mAb infusion blocked affinity maturation of the majority of the anti-Env CD4bs response,^13^^,^^15^^,^^16^^,^^22^ and allowed higher affinity maturation toward alternative epitopes.

Within the CD4bs specific mAbs, the K_D_ distribution was right-tailed following mAb infusion whereas it was log-Gaussian after the control immunization (p values of Shapiro-Wilks test on log2 transformed reciprocal K_D_ values; 4.427x10^−6^ and 0.7122 respectively). This observation is compatible with the emergence of a sub-population of B cells which can recognize the CD4bs with higher affinities following mAb infusion with respect to the remainder of the group and the infused antibodies. The divergence appears at approximately the K_D_ value of NIH45-46 (1.22x10^−8^M), and therefore may suggest that the few B cells that could survive the increased mAb selection pressure were able to reach equivalent/higher affinities than CD4bs B cells responding to Env gp120 immunization alone, as reflected in Figure 1D. Binding of most mAbs in this high affinity group (7 out of 8) was completely lost by the D368R mutation, whereas binding was only lost by 3 out of 9 higher affinity mAbs from the control immunization. This shows that these high affinity mAbs from the mAb infusion group would likely compete for Env gp120 binding with the infused NIH45-46 and PGV04 bnAbs, which are also dependent on the D368 residue (Figure S3;^23^). Other mAbs within this high affinity group, although still bound the CD4bs using the D368 residue, were less reliant on it, evidenced by a reduction in binding (knock down) rather than complete removal (knock out). Such mAbs were able to be identified due to the more permissive sorting strategy used in this study than alternative approaches.^24^ In summary, the B cell response to non-CD4bs epitopes can be generally enhanced by CD4bs-specific mAb infusion (Figure 1C). In contrast, the affinity of most CD4bs-specific mAbs is restrained by mAb infusion (Figure 1C) but there is a trend toward the development of a subset of higher affinity B cells within the CD4bs-specific response (Figure 1D).

### mAb infusion selects distinct CD4bs V_H_ genes and maintains diversity amongst the highest affinity B cells

**Figure 2:**
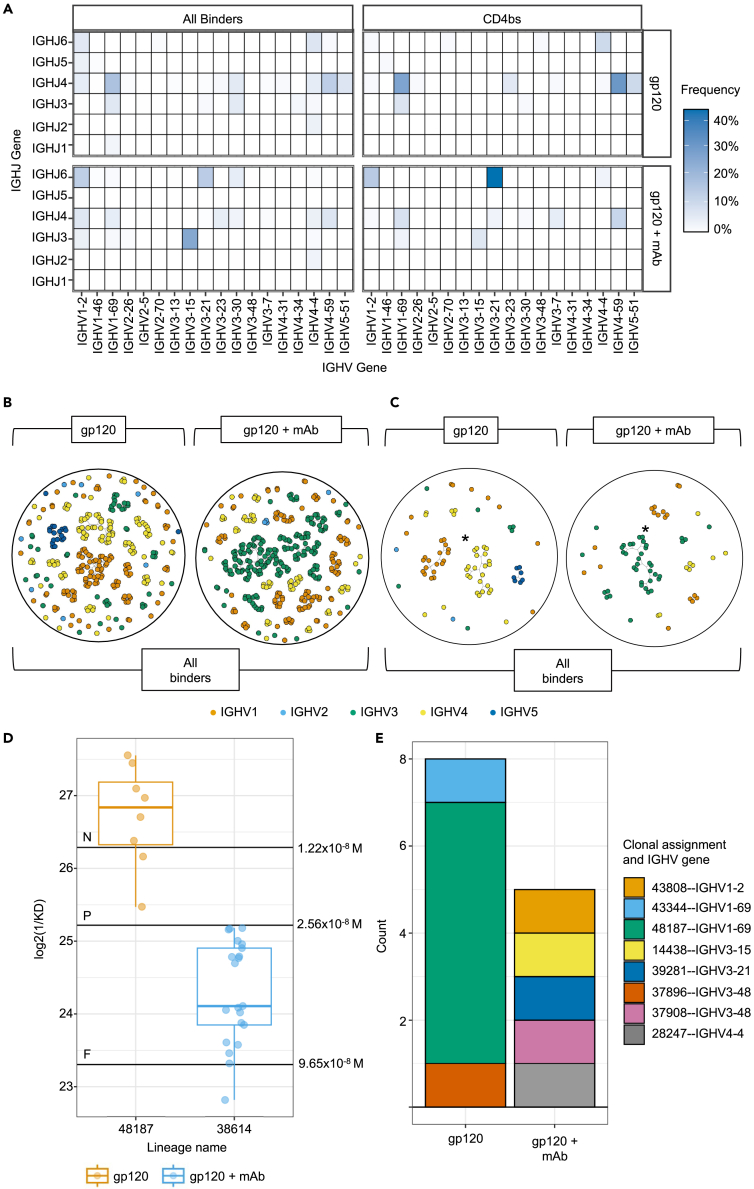
mAb infusion can expand an alternative V_H_ clonal lineage following repertoire pruning and may facilitate greater diversity in the highest affinity cells Analysis of V_H_J_H_ gene use ang clonal expansions within antigen specific B cells. (A) V_H_J_H_ gene usage frequency in whole gp120 binders (top) and CD4bs specific B-cells only (bottom). Color bar depicts the frequency within each immunization and epitope group. (B) Clonal expansion plots for all gp120 binders or (C) CD4bs-specific mAbs between different immunization regimens, colored by V_H_ gene family. Each point represents a V_H_ chain, and points are linked if the 2 B-cells are part of the same clonal group, based on having identical V_H_ & J_H_ genes, identical CDRH3 length, and 90% amino acid similarity within the CDRH3. Networks were created using the igraph R package. Indicated lineages analyzed in Figure 4D are marked with ‘∗’. (D) Log2 transformed reciprocal KD values for the dominant CD4bs specific clonal expansion in control immunization (clone ID: 48187) and mAb infusion immunizations (clone ID: 38614). The N, P and F annotations reflect the affinities of NIH45-46, PGV04, and F105 mAbs respectively. Boxplots illustrate the distribution of quantiles 1 and 3 (Q1 and Q3) with the horizontal line representing the median. Whiskers represent Q1 or Q3 +/- of the interquartile range. (E) V_H_ genes and clonal family names used by the mAbs in each immunization group that exceed the affinity of NIH45-46 (identified in Figure 3C).

To understand altered GC selection following mAb infusion we investigated the features of the V_H_ sequences of antigen-specific B cells. MAb DNA was sequenced and annotated for Ig gene features, such as the variable region genes used, and clustered into clonal lineages. The V_H_J_H_ gene combinations within the Env gp120-specific and CD4bs-specific mAbs were first assessed (Figure 2A), and displayed no consistent signature of V_H_J_H_ genes between the two regimens across either epitope group. Env gp120-specific cells in mAb infused mice were enriched for IGHV3-15—IGHJ3 (n = 86, 24.7%) and IGHV3-21—IGHJ6 (n = 47, 13.5%), whereas IGHV1-69—IGHJ4 (n = 47, 16.4%) and IGHV5-51—IGHJ4 (n = 19, 6.6%) were enriched in the control group. The relative frequency of B cells using the IGHV3-21—IGHJ6 recombination increased in mAb infused mice (n = 29, 44.6%) whereas the IGHV3-15—IGHJ3 recombination decreased (n = 4, 6.2%), with similar frequencies observed in the other V_H_J_H_ genes. For control immunized mice, IGHV1-69—IGHJ4 and IGHV5-51—IGHJ4 remained enriched (n = 19, 26.0% and n = 7, 10.0% respectively), with IGHV4-59—IGHJ4 (22, 30.1%) being more represented in CD4bs-specific B cells than in total Env gp120-specific B cells.

Env gp120-specific B cells derived from mice receiving mAb infusion were primarily comprised of two main clonal expansions (Figure 2B). These expansions use the IGHV3-15 and IGHV3-21 genes highlighted above, whereas control immunized mice had smaller clonal expansions using more diverse V_H_ family genes. Control mice also produced more singletons (cluster size = 1) than mice receiving mAb treatment (n = 84 and n = 43 respectively). Figure 2C illustrates clonal expansions for B cells which target the CD4bs. Here, control immunized mice have three main clonal expansions, comprised of the IGHV1-69—IGHJ4, IGHV5-51—IGHJ4, and IGHV4-59—IGHJ4, whereas clonal expansion following mAb infusion is dominated by one IGHV3-21—IGHJ6 lineage. A range of VH genes for CD4bs cells were isolated for both immunization regimens, rather than the stereotypical IGHV1-2 gene found within human CD4bs neutralizing response (*20*). Clonal imbalance was quantified by Gini index (Figure S4), which shows significantly lower clonal and V_H_J_H_ gene usage dominance in Env gp120 specific B cells following control immunization, however this was the only group that was statistically different. The decision to pool tissues from multiple mice limited the direct interpretations of B cell clonality, however the use of bulk sequencing data found that most B cell families were derived from a single mouse (Table S1), and thus most of the displayed lineages were likely from single mice, although this cannot be conclusively proved.

We next compared the K_D_ values of mAbs making up the dominant CD4bs clonal expansion in each immunization group (Figure 2D), signified with an asterisk in Figure 2C. The lineage IDs were 38614 from mAb infused mice (IGHV3-21—IGHJ6) and 48187 from control immunized mice (IGHV1-69—IGHJ4). We observed that in these similarly sized clonal expansions, all members of lineage 38614 were of lower affinity than lineage 48187. Interestingly, no members of lineage 38164 were able to surpass the K_D_ of PGV04, the second mAb infused (2.56x10^−8^M). No such upper limit or ceiling was present for lineage 48187 from control immunized mice, with K_D_ values ranging from 5.1nM to 21.5nM and possessed a continuum of affinity values throughout the lineage. Given that the distribution of the affinities for lineage 38614 was less continuous, and several non-AA sequence redundant members were unable to surpass the affinity of PGV04, we hypothesize that the infused mAbs applied an affinity ceiling which was unable to be surpassed by this lineage. This data suggests that initial clonal expansions targeting the CD4bs in response to Env gp120 immunization may have been prevented from further participation in the GC by elevated competition from the infused mAbs.

Finally, the V_H_ genes and clonal assignments in mAbs whose K_D_ surpassed NIH45-46 were considered (Figure 2E). Three such clonal lineages were present in control immunized mice, one of which used IGHV1-69 and comprised 75% (6 out of 8) sequences within the high affinity group. Interestingly, when considering mAbs with higher K_D_ than NIH45-46 in the mAb infused group, all sequences were derived from separate lineages that did not share any common V_H_ genes. Therefore, despite the higher selection pressure our data suggest was exerted by mAb infusion, the total diversity within high affinity CD4bs-specific B cells across the immunization regimen was greater under these conditions. Mining bulk plasma cell sequencing data for these lineages (Table S1) illustrates that these are sequences were probably from a range of mice. Lineage 43808 identified in Figure 2E used IGHV1-2 in its variable region, however its light chain CDR3 length was 9 AAs, and thus too long to be VRC01 class.^25^ In summary, mAb infusion selected clonal families using different germline V_H_ genes to control (Figures 2A–2C) and was able to maintain more diversity within the highest affinity cells (Figure 2E).

### SHM is reduced in Env gp120 specific B cells following mAb infusion, except in the highest affinity CD4bs specific cells

**Figure 3:**
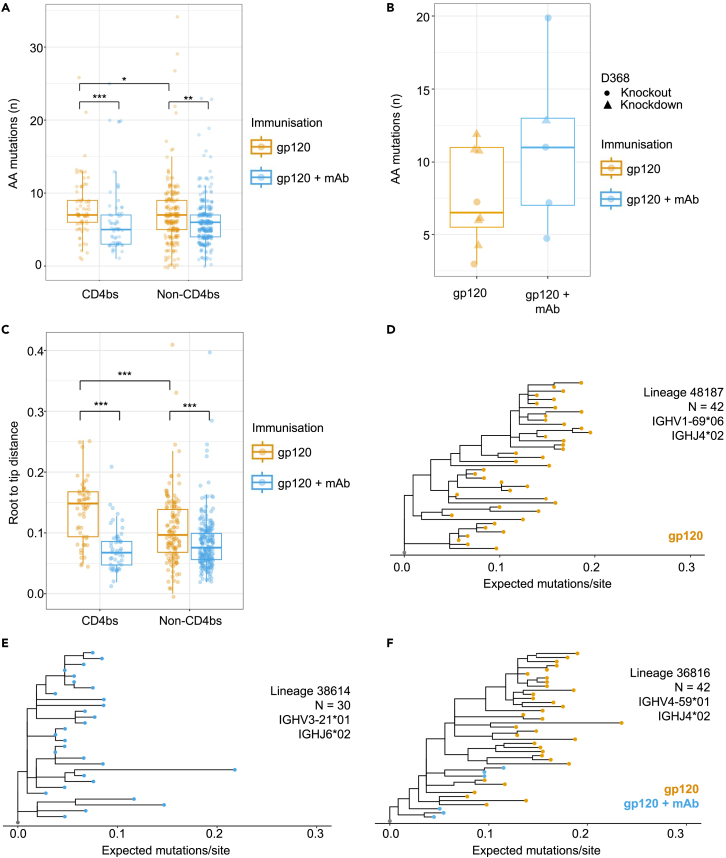
mAb infusion reduces SHM in most Env gp120 specific mAbs Analysis of SHM in Env gp120 specific B cells. (A) Number of amino acid mutations across the entire V_H_ sequence between different epitope groups and immunization regimens, calculated using the observedMutations function in the shazam R package. Mann-Whitney test p-values: gp120-CD4bs vs gp120 + mAb-CD4bs: p = 0.0002, gp120-Non CD4bs vs gp120 + mAb-Non CD4bs: p = 0.0023, gp120-CD4bs vs gp120-Non CD4bs: p = 0.0131, gp120 + mAb-CD4bs, gp120 + mAb-Non CD4bs: p = 0.3373). (B) Number of amino acid mutations across the entire V_H_ sequence in the CD4bs-specific mAbs whose affinities exceed NIH45-46 within the different immunization regimens. Mutations were calculated as in A (Mann-Whitney test p-value = 0.2377). Circular points reflect mAbs whose binding is knocked out by the D368R mutation, and triangular points reflect mAbs whose K_D_ is reduced by at least 10-fold. (C) Root to tip distances calculated using the distRoot function from the adephylo R package. Trees were first estimated using IgPhyML, before using the sequence identifier to map the epitope specificity. Mann-Whitney test p-values: gp120-CD4bs vs gp120 + mAb-CD4bs: p = 8.784x10^-10^, gp120-Non CD4bs vs gp120 + mAb-Non CD4bs: p = 1.235x10^-5^, gp120-CD4bs vs gp120-Non CD4bs: p = 4.535x10^-5^, gp120 + mAb-CD4bs vs gp120 + mAb-Non CD4bs: p = 0.1476. (D) Representative phylogenetic trees from Env gp120 only immunization. (E) Representative phylogenetic trees from Env gp120 immunization with mAb infusion. (F) Representative phylogenetic trees from a convergent lineage present in both immunization groups. Tips are colored by immunization regimen, except for the inferred germline node (gray). Boxplots illustrate the distribution of quantiles 1 and 3 (Q1 and Q3) with the horizontal line representing the median. Whiskers represent Q1 and Q3 +/- of the interquartile range.

Given that alternative V_H_ recombinations were identified following mAb infusion, the question arose whether the degree of SHM within the V_H_ also differed (Figures 3A–3F). Figure 3A shows that there are significantly fewer AA mutations within CD4bs-specific B cells following mAb infusion than after control immunization (p = 0.0002). This agrees with the affinity data (Figure 1C) and corroborates that CD4bs B cells are mostly excluded from the response by the high affinity mAb infusion. Mice from the control immunization group had significantly more mutations in their non-CD4bs mAbs than mice experiencing mAb infusion, despite these control non-CD4bs mAbs having slightly lower affinity. Since non-CD4bs mAbs target many diverse epitopes, different mutation frequencies can produce mAbs with a range of affinity for antigen, depending on which epitope is being targeted. Figure 3B presents the SHM levels within the most affinity matured CD4bs mAbs (identified in Figure 1D). Since we focus on such extremes of affinity maturation, there are too few mAbs meeting these criteria from each group to obtain a statistically significant result (p ≥ 0.05). However, we observed a trend toward having greater numbers of mutations following mAb infusion in the high affinity subset. This complements the affinity data presented in Figure 1D, and reinforces the idea that a small portion of CD4bs B cells can acquire affinity enhancing mutations due to the elevated selection pressure in the presence of infused CD4bs bnAbs. Members of such B cell lineages may therefore be enriched in the bone marrow plasma cell repertoire, since these canonically possess the highest affinity antibodies.

Next, to assess the evolution of B cells participating in the anti-Env response, all Env gp120 binding lineages with at least 5 members were considered for phylogenetic analysis using IgPhyML. Figure 3C demonstrates the root to tip distance (i.e., distance from the inferred germline to each observed sequence in the lineage). The differences between the groups reflect the observed for the number of AA mutations shown in Figure 3A, albeit with more pronounced changes. This is illustrated graphically in the representative lineages displayed in Figures 3D–3F. Figures 3D and 3E present lineages 48187 and 38614, from control and mAb infused immunization regimens respectively, initially highlighted in Figures 2C and 2D. Lineage 48187, containing some of the highest affinity mAbs from control immunization, has a more branched structure than lineage 38614 which is suggestive of more structured antigen-specific evolution.^26^ This correlates with the affinities of these two lineages displayed in Figure 2D, where lineage 38614 appeared to be restricted in its affinity maturation by the mAb PGV04. These phylogenetic trees were derived from the pooled antigen-specific splenocytes, however bulk sequencing information (Table S1) demonstrates that lineage 48187 (Figure 3D) was found only in one mouse and the presented tree is therefore highly likely to be derived from one mouse. Lineage 38614 was present in several mice from the mAb infusion immunization, however was strongly enriched (≥90% of members) in a single mouse. As a result, this phylogenetic tree predominantly illustrates the evolution of this lineage in a single mouse. Additionally, Figure 3F illustrates a convergent lineage shared between both immunization regimens by multiple mice. Within this lineage, members derived from the mAb infusion immunization lie closer to the germline sequence than those from the Env gp120 only immunization. While this is not a true biological lineage, since lineage members are derived from different mice, it illustrates how a common B cell precursor could evolve differently due to the different immunization regimens. Full information on all lineages analyzed is present in Table S2. In summary, these data highlight that SHM in most CD4bs B cells is reduced after the mAb infusion immunization (Figures 3A and 3C–3F), however infused mAb pressure may allow elevated SHM in a restricted set of higher affinity B cell lineages (Figure 3B).

### CD4bs-specific bone marrow plasma cells are significantly more mutated following mAb infusion

Thus far, BCR sequence data were obtained exclusively from antigen specific B cells isolated from lymph node and splenic tissues. However, during the humoral immune response, the most affinity matured B cells differentiate into plasma cells and exit from the secondary lymphoid organs to reside in the bone marrow.^3^^,^^27^ Since plasma cells down-regulate surface Ig, antigen-specific PCs cannot be isolated with traditional single cell sorting. Therefore, plasma cells were enriched using magnetic beads from bone marrow tissues, and bulk next generation sequencing (NGS) was carried out on V_H_ mRNA derived from IgG expressing cells. Epitope specificity was assigned based on CDRH3 clustering with V_H_ sequences from the experimentally validated mAbs that were isolated by cell sorting. NGS-derived sequences that clustered with sequences from FACS-derived lineages containing at least 33% CD4bs-specific members were defined as CD4bs specific. This threshold represented the median frequency of mAbs assigned as CD4bs within all experimentally tested lineages and allowed selection of 25% of the CD4bs lineages discovered using single cell sorting. mAbs from these lineages, derived from bone marrow tissue, were termed putative CD4bs-specific.

**Figure 4:**
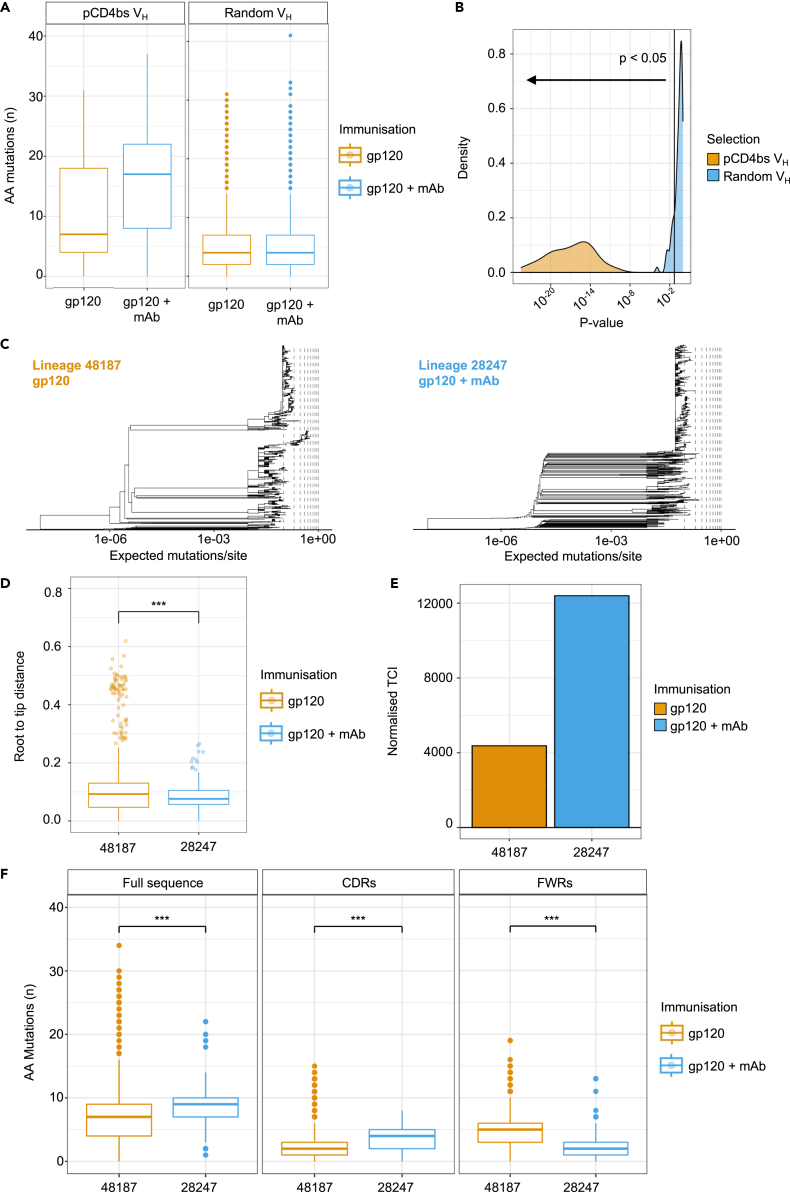
Putative CD4bs bone marrow plasma cells are significantly more mutated following immunization with mAb infusion Analysis of IgG V_H_ sequences derived from magnetic enriched bone marrow plasma cells. In the absence of direct antigen specific information, sequences identified in the bone marrow were clustered with sequences from lymph nodes. Sequences that clustered with lymph node lineages with at least 33% of members targeting the CD4bs were assigned as putative CD4bs specific. (A) Comparison of amino acid mutations between a random selection of 2000 sequences per immunization group (left) or the putative CD4bs binding lineages per immunization group (right); gp120 immunization (n = 6627), gp120 + mAb immunization (n = 3302). (B) Significance was assessed through repeat resampling (with replacement) of 500 sequences from the either the putative CD4bs lineages, or all lineages (similarly to A). Within these samples, a Mann-Whitney test was carried out on the number of amino acid mutations per sequence between the immunization regimens. This process was repeated 100 times, storing the p value on each iteration. The distribution of these p values per group is on the density histogram in this panel. The vertical line signifies p = 0.05, and deviation toward the left indicates statistically significant changes in the data. (C) Maximum likelihood phylogenetic trees for selected lineages (including data from low throughput sequencing) from each immunization regimen. Prior to estimating the trees, single reads were removed and identical sequences were collapsed, prior to randomly selecting 1000 sequences per lineage. Phylogenetic tree x axis is displayed on a log10 transformed scale to highlight earlier branching events. Vertical lines reflect incremental increases of 0.1 expected mutations per site, beginning at 0.1 prior to axis transformation. (D) Root to tip distances calculated using the distRoot function from the adephylo R package, for the two lineages under investigation (control; 48187, mAb infusion; 28247). Measurement is performed on the trees estimated in C, and values outside 1.5 x the interquartile range are displayed as points. Mann-Whitney p-value: 9.598 x 10^-16^. (E) Total cophenetic index for the lineages under investigation, normalized by the number of tips per tree, to demonstrate differences in phylogenetic tree balance. (F) Number of amino acid sequences across the entire V_H_ sequence (left), CDRs only (middle) or FWRs (right), of pCD4bs lineages 48187 and 28247 from control and mAb infused mice respectively. Mutations are counted as per Figure 5A. Mann-Whitney test p-values (left to right): 0.0006, 1.071 x 10^-6^, 6.097 x 10^-16^. All boxplots illustrate the distribution of quantiles 1 and 3 (Q1 and Q3) with the the horizontal line representing the median. Whiskers represent Q1 and Q3 +/- of the interquartile range.

Firstly, to assess if there was more affinity maturation in putative CD4bs sequences after mAb infusion, the number of AA mutations per V_H_ was measured in both putative CD4bs and randomly sampled V_H_ sequences (Figures 4A and 4B). AA mutations were significantly higher in putative CD4bs plasma cells after mAb infusion. In contrast, single CD4bs B cells from the secondary lymphoid organs demonstrated significantly lower SHM (Figure 3). This suggests that the most affinity matured members of these CD4bs-specific lineages exited from the GC and migrated to the bone marrow. The elevated AA mutation frequency was not a general feature of bone marrow plasma cells after mAb infusion, since the randomly sampled sequences were equally mutated between immunization regimens.

**Figure 5:**
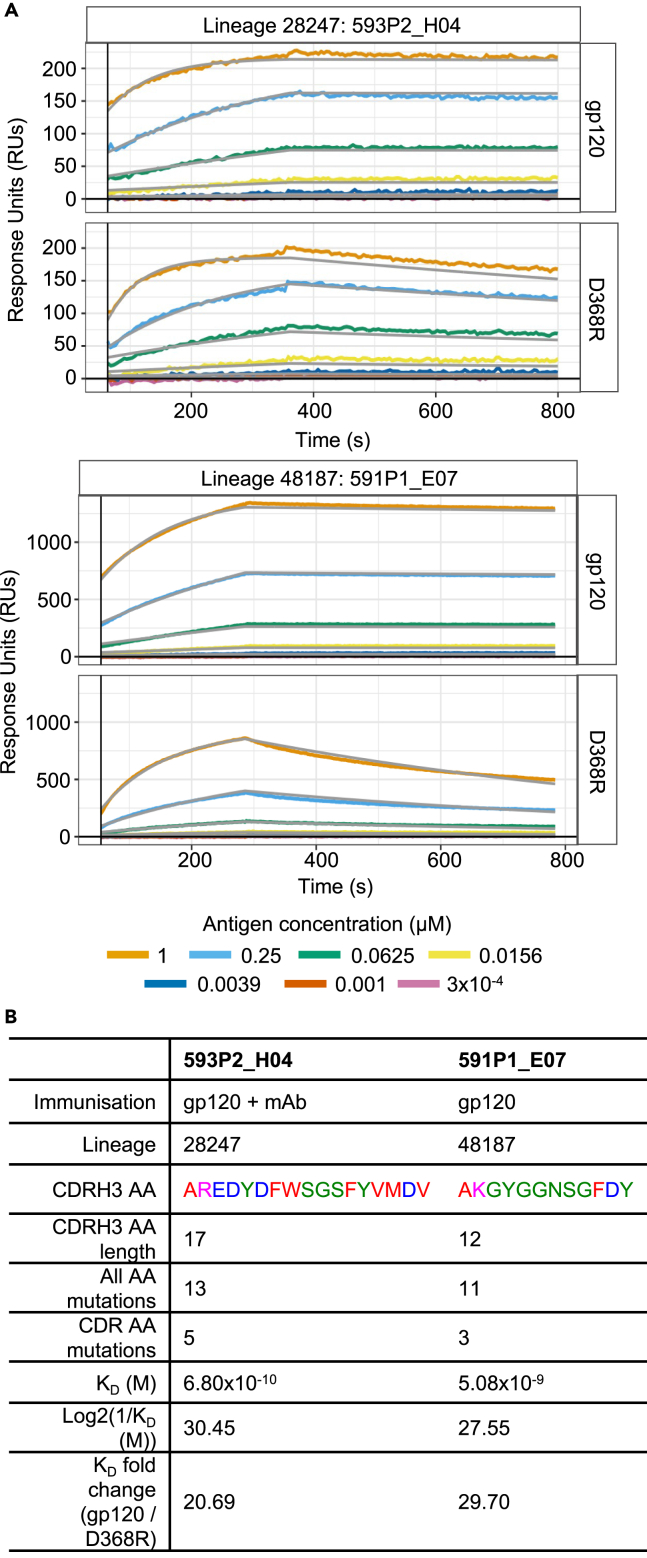
The highest affinity GC B cell from the representative lineages underwent more affinity maturation following mAb infusion The highest affinity member of lineage 28247 (593P2_H04) and lineage 48187 (591P1_E07), identified by FACS and Sanger sequencing, were further investigated for affinity maturation. (A) Sensorgrams depicting binding affinity of the aforementioned mAbs to MGRM8 Env gp120 (top) and MGRM8 D368R Env gp120 (bottom). (B) Table reflecting the maturation characteristics of both members.

We next characterized putative CD4bs lineages from both mAb infused and control immunized mice phylogenetically (Figures 4C–4E). A representative lineage was selected from each immunisation group, namely lineage 48187 and lineage 28247 from control and mAb infusion immunizations respectively. These lineages were selected since they were large (n ≥ 1000 unique nucleotide sequences), similarly sized, and at least one of their members from low-throughput sequencing had higher K_D_ values than NIH45-46 (Figure 2E). Phylogenetic tree topology was investigated in Figure 4C. Lineage 28247 (right) presents a shorter tree with many early branching events, whereas the lineage 48187 tree (left) is longer overall, with outgrowth of a subtree toward the center. Lineage 48187 presents significantly greater distance from germline than lineage 28247, quantified by root to tip distance (p = 9.6x10^−16^, Figure 4D). Tree imbalance was also measured by using the total cophenetic index (TCI), normalized by the number of tips per tree (Figure 4E). This shows that lineage 28247 is more imbalanced than lineage 48187 despite its tree being shorter, which is likely related to the rapid early branching events visualized in Figure 4C. The shorter, earlier diversifying phylogenetic tree from lineage 28247 plasma cells suggests that their precursors spent less time within the GC before differentiating. Interestingly, this is not reflected in the total number of AA mutations present, since these were significantly higher in lineage 28247, with most mutations present in the CDRs (p ≤ 2.2x10^−16^ for both whole sequence and CDR comparisons; Figure 4F, left and center). Lineage 48187 presented more mutations in the FWRs (Figure 4F, right; p ≤ 2.2x10^−16^). mAb infusion therefore appears to have allowed more rapid SHM than control that focused on the primary antigen binding regions within these example lineages. This finding prompted analysis of the highest affinity member from these lineages using the data from the lymph nodes, revealing that lineage 28247 achieved more AA mutations (13 vs. 11) and almost 10-fold higher affinity (0.07x10^−8^M and 0.51x10^−8^M) in its most affinity matured member than lineage 48187 (Figure 5). Therefore, mAb infusion during the immunization appeared to both accelerate the development of lineage 28247, whilst allowing its maximum affinity to reach higher levels than was possible in a similar lineage from the control immunization.

In summary, despite CD4bs V_H_ sequences incurring less SHM in peripheral lymphoid tissues, putative CD4bs bone marrow plasma cells incurred significantly more SHM (Figures 4A and 4B). A relevant large lineage from each immunization group demonstrated more mutations accrued after what appeared to be less time spent in the GC (Figures 4C–4G), suggestive of more efficient SHM. The speed of this evolution correlated with the affinity of the highest affinity lineage member. Repertoire pruning, as in the sequential infusion of escalating affinity mAbs described here, therefore appears to have allowed more rapid and stronger maturation of a selected group of B cells.

## Discussion

The importance of soluble antibody in B cell responses has long been established,^10^^,^^12^^,^^14^^,^^18^ and this study provides additional insight into how this phenomenon can be leveraged. Through our repertoire pruning approach, we describe an immunization strategy designed to overcome the previously observed negative effects of mAb infusion on the post-GC affinity. When considering all CD4bs-specific B cells, we show that presence of high affinity competing antibody results in reduced affinity maturation when considering key measures including lower BCR affinity, reduced SHM, and shorter phylogenetic distance from germline. This result is consistent with recent works investigating GC responses and antibody affinity.^11^^,^^13^^,^^14^^,^^15^^,^^16^^,^^18^^,^^22^ However, despite an overall decrease in CD4bs-specific affinity maturation, we identified a sub-population of CD4bs B cells whose affinity exceeded that of the highest affinity mAb infused (K_D_ ≥1.22x10^−8^ M). Moreover, this sub-population had a higher average affinity in the mAb infusion group than the control group, with concurrent greater SHM. One such lineage (28247) was also discovered within the bone marrow plasma cell repertoire and had accrued significantly more CDR mutations more rapidly than a relevant comparison lineage from control immunization (48187). Lineage 48187 also had more FWR mutations than lineage 28247. Since these regions are primarily structural,^26^ it suggests that either non-affinity enhancing mutations were permitted in the control lineage, or that subtle changes in the antibody structure were required to complement the CDR mutations. Taken together, this suggests that SHM was more efficient using repertoire pruning, and greater affinity mAbs can be generated in less time. Longitudinal sequencing, which would be better able to analyze the faster development of high affinity CD4bs-specific B cells following mAb infusion, was not possible in this study.^26^^,^^28^^,^^29^ Since this study’s application lies in therapeutic mAb generation, B cell isolation from each mouse was only possible at the end of the immunization regimen. However, a role for soluble antibodies in modulation of selection was identified in our proof of principle study, and future studies that harvest tissues from subsets of mice during an immunization regimen could be used to obtain longitudinal data from a significantly larger cohort of mice. Such experiments could shed light on the complex B cell repertoire dynamics in the setting of multiple immunizations, which has not yet been carried out in antibody feedback studies. However, interpretation of this data would be limited by the repertoire diversity observed in different mice in response to the same immunogen. It would also rely on the presence of sufficient convergent lineages across the mice, which, despite being observed in higher frequencies than initially believed,^29^^,^^30^^,^^31^^,^^32^ still represent a minority of the B cell repertoire.

**Figure 6:**
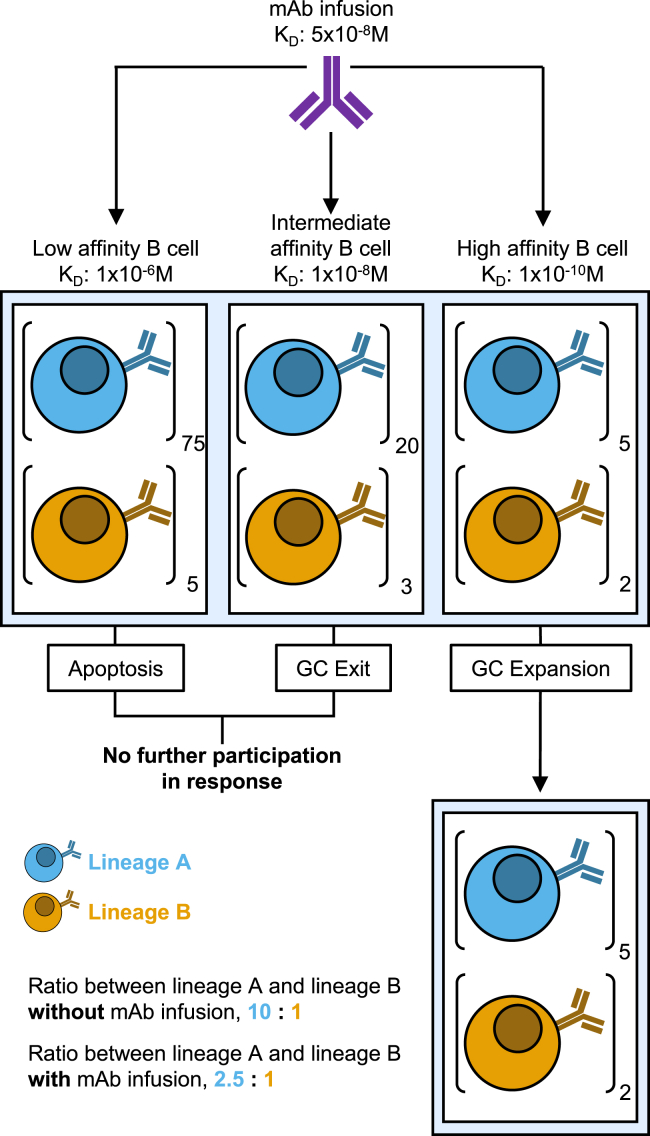
Proposed mechanism of diversity enhancement by mAb infusion Schematic for how mAb infusion may enforce greater diversity within the highest affinity B cell populations. This diagram represents two lineages, A (blue) and B (yellow), within a GC which have similar affinity distributions but different total sizes. The initial ratio between these two lineages was 10:1 in favor of lineage A. Upon application of a high affinity soluble antibody only those clonal members of A and B that have higher affinity than infused mAb (right panel) are selected. Thus, the ratio of the A and B lineage members that can respond now shrinks to 2.5:1. Therefore, through inducing apoptosis or GC exit in lower affinity B cells within binding lineages, mAb infusion may standardize the frequencies of the lineages meeting the selection criteria and therefore allow selection for a broader range of B cell lineages.

Prior literature that reported suppression of the epitope specific response by mAbs overwhelmingly involved either pre-treatment or co-administration of high affinity mAbs during the immunisation.^8^^,^^10^^,^^14^^,^^15^^,^^18^ This likely applied extreme selection pressure to naive B cells and prevented their recruitment into the GC. Instead, we opted for raising selection pressure more gently by performing the first immunization without exogenous antibody pressure, to recruit naive cells uninhibited, and later by infusing mAbs of escalating affinity after each dose. This allowed iterative expansion followed by suppression of the CD4bs-specific B cell response and produced the small panel of higher affinity B cells observed here. Interestingly, these high affinity cells also made use of a more diverse range of V_H_ genes. In accordance with the elevated GC apoptosis noted following mAb infusion^12^ as well as the modulation of GC fate decisions by antigen availability,^5^^,^^33^ reduction in antigen binding by LZ B cells due to mAb infusion likely induced apoptosis or GC exit in lower affinity CD4bs-specific B cells during our study. We propose that by forcing earlier GC exit of these low-to-intermediate affinity lineage members, mAb infusion allowed continued GC participation of only the highest affinity B cells, thereby balancing frequencies and facilitating selection of a wider range of lineages (Figure 6). Therefore, despite the number of high affinity antibodies discovered being smaller, repertoire pruning generated high affinity epitope-specific mAbs with greater genetic diversity across the mAb infusion immunization regimen. We predict that this increases the likelihood that the antibodies isolated from the mAb immunization regimen represent a range of binding modalities and functions, compared to the oligoclonal expansion from the control immunization.^34^^,^^35^ Furthermore, whilst lower affinity CD4bs-specific B cells are prevented from participating in the GC due to mAb infusion occluding their epitope, no such suppression would be present for non-CD4bs-specific B cells. In fact, by reducing the participation of CD4bs-specific B cells, the selection of non-CD4bs cells may be enhanced by allowing them greater access to antigen captured on the FDC surface.^36^^,^^37^ This would translate to delivery of stronger proliferative signals during positive selection by Tfh cells^6^^,^^38^ and would result in the affinity enhancement of non-CD4bs specific cells that was observed in this study. Therefore, the positive effects of mAb infusion likely result from a concert of increased selection pressure and elevated antigen availability for B cells of multiple specificities, rather than from a single reason. However, these conclusions would be more easily enforced were the tissues retained separately per immunization regimen rather than being pooled.

Such observations are potentially useful for understanding the role of soluble mAbs during HIV infection, given our model antigen was HIV Env gp120. Our data suggests that infusion of antibody could modulate HIV-specific B cell responses, but we cannot infer if this would be positive or negative for the overall response, since the dominant effect of our mAb immunization regimen was suppression of competing B cell affinity maturation. For example, in prior reports, the CH103 CD4bs bnAb lineage was suggested to be triggered following viral escape from a non-neutralizing antibody lineage.^39^ Similarly, viral escape from 3BNC117 infusion was seen to increase serum neutralising breadth in people living with HIV.^40^ These publications show that infusion of Env binding mAbs could improve bnAb generation during infection by forcing viral escape mutants that are more readily neutralized. Indeed, an improvement in endogenous antibody responses have been observed in some bnAb therapy studies.^41^ Our data suggests that the presence of soluble antibody may also enhance affinity maturation in certain lineages leading to some higher affinity mAbs, but not improving the total serum response. Therefore, passive mAb infusion during HIV infection would function to both diversify and affinity mature the response. Since we used a protein-based immunization rather than a viral challenge, we were unable to measure this in our study. Moreover, we would like to note that these are suggestions based on available data in other studies and the research we present here, rather than directly inferred from our work.

In summary, this study highlights a method for improving the mAb discovery process by applying increasingly strong antibody feedback to influence selection of B cells within the GC. Use of this repertoire pruning method within mAb discovery pipelines could enable enhanced mAb affinity without lengthy post- immunization selection methods, thus streamlining drug discovery timelines. *In vitro* affinity maturation and selection using phage display techniques may take several months to engineer a higher affinity variant, therefore achieving diverse antibodies of higher affinity in a single immunization regimen is of critical importance. A further application of our findings is to use mAb infusion to suppress immunodominant responses during vaccination, while enhancing affinity toward alternative epitopes. Theoretically, this would allow faster affinity maturation to sub-immunodominant epitopes, without the additional immunization previously seen to saturate the immunodominant epitopes.^13^ This has been shown in a serum transfer experiment in mice. Here, transfer of anti-SARS-CoV-2 Wuhan strain serum was able to suppress primary responses to variable epitopes within SARS-CoV-2 Wuhan strain, while increasing responses to epitopes conserved between Wuhan and Omicron stains.^22^ In combination with our study, this suggests that mAbs can reduce responses to target regions which could facilitate broader immunity. Overall, these data support the affinity-guided use of mAb infusion during immunization to induce stronger selection pressure for higher affinity clones and promote the rapid formation of antigen-specific bone marrow plasma cells.

### Limitations of the study

The main limitation of this study was the decision to pool murine spleen and LN tissues prior to antigen-specific cell sorting. This decision was made to allow greater numbers of antigen-specific sequences to be processed during cell sorting, however it impacted upon conclusions by reducing the number of biological replicates available. Bone marrow tissues were retained separately for each mouse, and high-throughput sequencing on these tissues was able to deconvolute the likely sources of each B cell lineage. It is believed that this provided an accurate solution, however the decision to pool mice limited insights into reproducibility of the effect postulated within this report. A further limitation is that it is unknown whether a lower dose of the high affinity antibodies could enhance epitope specific B cell maturation more broadly, rather than limiting it to the population identified in this study. Although out of scope for our study, this would be best achieved through a comprehensive dose finding experiment, with a matrix of antibody doses infused across all timepoints.

## STAR★Methods

### Key resources table

**Table:** 

REAGENT or RESOURCE	SOURCE	IDENTIFIER
**Antibodies**		

Alkaline Phosphatase-Conjugated Goat anti-mouse IgG	Jackson Immunoresearch	Cat #115-055-071 RRID: AB_2338535
Alkaline Phosphatase-Conjugated goat anti-human IgG	Jackson Immunoresearch	Cat #109-055-098 RRID: AB_2337608
Horse radish peroxidase-conjugated goat anti-mouse IgG Fc	Bio-rad	Cat # STAR120 RRID: AB_567024
Anti-mouse Ter119 PerCP-Cy5.5	BD Biosciences	Cat #560512 RRID: AB_10561844
Anti-mouse CD3 PerCP-Cy5.5	Biolegend	Cat #300430 RRID: AB_893299
Anti-mouse Ly6G PerCP-Cy5.5	BD Biosciences	Cat #560602 RRID: AB_1727563
Anti-mouse CD11c PerCP-Cy5.5	BD Biosciences	Cat #560584 RRID: AB_1727422
Anti-mouse B220 PE-Cy7	Biolegend	Cat #103222 RRID: AB_313005
Anti-mouse IgG BV421	Biolegend	Cat #405317 RRID: AB_10900419
Anti-mouse IgM BV510	Biolegend	Cat #406531 RRID: AB_2650758
Biontinylated Anti-mouse Ter119	StemCell	Cat #100-0456 RRID: *Awaiting RRID from Antibody registry*
Biotinylated Anti-mouse CD11b	Biolegend	Cat #101204 RRID: AB_312787
Biotinylated Anti-mouse CD71	Biolegend	Cat #113803 RRID: AB_313564
Biotinylated Anti-mouse CD105	Biolegend	Cat #120404 RRID: AB_961062
Biotinylated Anti-mouse CD3	Biolegend	Cat #100244 RRID: AB_2563947
Biotinylated Anti-mouse CD11c	eBioscience	Cat #13-0114-82 RRID: AB_466363
Biotinylated Anti-mouse CD34	Biolegend	Cat #128604 RRID: AB_1236371
Biotinylated Anti-mouse CD5	Biolegend	Cat #100604 RRID: AB_312733
Biotinylated Anti-mouse IgD	Southern Biotech	Cat #1120-08 RRID: AB_2631189
Biotinylated Anti-mouse IgM	Biolegend	Cat #406504 RRID: AB_315054
Biotinylated Anti-mouse CD49	Biolegend	Cat #103604 RRID: AB_313035
Murine Fc Block	Biolegend	Cat #: 101320 RRID: AB_1574975

**Chemicals, peptides and recombinant proteins**		

MGRM8 Env gp120	This paper and (Suzuki et al.^37^)	N/A
MGRM8 D368R Env gp120	This paper	N/A
BG505 D368R Env gp120	This paper	N/A
PEI-MAX (40K)	Polysciences, Inc	Cat #24765-1
Streptavidin-Conjugated phycoerythrin	Invitrogen	Cat #S21388
Streptavidin-Conjugated Allophycocyanine	Invitrogen	Cat #S32362
Near IR Dead Cell Stain	Invitrogen	Cat #L10119

**Critical commercial assays**		

QuantTag Biotin Quantification kit	2B Scientific	Cat #BDK-2000
QuikChange Lightning SDM kit	Agilent	Cat #210518
ExpiFectamine Transfection kit	Gibco	Cat #31985062
NEBuilder HiFi DNA Assembly kit	New England Biolabs	Cat #E5520S
EZ-Link™ Sulfo-NHS-LC-Biotin kit	Thermo Scientific	Cat #21335
QIAcuity EG PCR Kit	QIAGEN	Cat #250111

**Deposited data**		

BCR Sequencing data: Antigen specific (Sanger) V_H_ sequences	This paper; Mendeley Data	Accession: Mendeley Data: https://doi.org/10.17632/5k5thsgxw7.1
BCR Sequencing data: Antigen specific (Sanger) V_K_ sequences	This paper; Mendeley Data	Accession: Mendeley Data: https://doi.org/10.17632/5k5thsgxw7.1
BCR Sequencing data: Plasma cell enriched bulk IgG	This paper; Mendeley Data	Accession: Mendeley Data: https://doi.org/10.17632/5k5thsgxw7.1
Code to generate BCR networks	This paper; Github	Accession: Github: https://github.com/pthomas92/CD4bs-mAb-Infusion/tree/main

**Experimental models: Cell lines**		

Freestyle 293F Cells	Gibco	Cat #R79007
Expi293F cells	Gibco	Cat #A14527

**Recombinant DNA**		

Plasmid: MGRM8 Env gp120	This paper and (Voss et al.^42^)	N/A
Plasmid: MGRM8 D368R Env gp120	This paper	N/A
Plasmid: F105 mAb muFC	This paper	N/A
Plasmid: PGV04 mAb muFc	This paper	N/A
Plasmid: NIH45-46 mAb muFC	This paper	N/A

**Software and algorithms**		

R v4.1.2	R Core Team (^49^)	https://www.r-project.org
Immcantation v4.4.0	Vander Heiden et al.,^55^) and Gupta et al., (^57^)	https://immcantation.readthedocs.io/en/stable/
FlowJo v10.8.2	BD Biosciences	https://www.flowjo.com
Chimera X v1.16	Petterson et al.*,* (^43^)	https://www.cgl.ucsf.edu/chimerax/
Tidyverse v1.3.2	Wickham et al., (^58^)	https://www.tidyverse.org/
Igraph v1.3.5	Csardi et al.*,* (^59^)	https://igraph.org

**Other**		

SuperScript III™	Invitrogen	Cat #N8080127
RNaseOUT™	Invitrogen	Cat #10777019
SuperScript IV™	Invitrogen	Cat #18091200
AMPure XP Magnetic Beads	Beckman Coulter	Cat #A63881
Q5 Hot-Start High Fidelity 2X Mastermix	New England Biolabs	Cat #M0494L

### Resource availability

#### Lead contact

More information and requests for reagents should be directed to and will be fulfilled by the lead contact, Laura E McCoy (l.mccoy@ucl.ac.uk).

#### Materials availability

This study did not generate unique resources or materials.

#### Data and code availability

BCR sequencing data has been deposited into Mendeley data (https://data.mendeley.com) and is publicly available from the date of publication (accession: https://doi.org/10.17632/5k5thsgxw7.1) also listed in the key resources table). Code used to generate BCR clonal networks in Figures 2B and 2C has been deposited into github and is publicly available as of the date of publication (https://github.com/pthomas92/CD4bs-mAb-Infusion/tree/main, also listed in the key resources table). Any additional information required to reanalyse the data reported in this paper is available from the lead contact upon request.

### Experimental model and study participant details

#### Ethics statement

All animal studies were ethically reviewed and carried out in accordance with Animals (Scientific Procedures) Act 1986 and the GSK Policy on the Care, Welfare and Treatment of Animals. An even mixture of male and female transgenic mouse pups were randomly selected within for each immunisation group, and had not received prior immunisation or treatments before this study.

### Method details

#### Study design

This study was designed to assess if infusion of escalating affinity epitope-specific mAbs could selectively enhance the affinity of competing B cells. To this end, two groups of transgenic mice containing human immunoglobulin loci were immunised with multiple doses of the MGRM8 Env gp120. One group also received repeated infusions of mAbs which targeted the CD4bs and incrementally increased in affinity with each infusion. Following the immunisation regimen, lymph node tissues were pooled, and antigen-specific B cells were isolated by single cell sorting from both immunisation groups. mAb encoding V_H_V_K_ mRNA was then recovered by single cell RT-PCR, which was used to synthesise mAbs for high-throughput experimental characterisation by SPR. Recovered sequences were also analysed for evidence of differential maturation due to the infused mAbs, using total AA mutation counts and phylogenetic assessments. Next, IgG sequences from bone marrow plasma cells were recovered by bulk NGS, and the antigen-specific B cells recovered previously were used to identify putative CD4bs-specific plasma cells. These plasma cells were analysed for evidence of affinity maturation using total AA mutation counts. Of these plasma cells, a representative highly expanded lineage per immunisation group was identified and analysed phylogenetically to assess evolution rates. Lastly, B cells clonally related to the representative plasma cell lineages were investigated to correlate mAb affinity with phylogenetic signatures.

#### MGRM8 Env gp120 generation

The plasmid encoding MGRM8 Env gp120 (GenBank accession number: MF510462, has been previously characterised^42^^,^^43^), and the D368R mutant version was generated using the Agilent QuikChange Lightning SDM kit (Agilent Technologies), using manufacturers recommendations. HEK293F cells for transfection were maintained in FreeStyle 293 culture medium (Gibco), at 37°C with 8% CO_2_ with an orbital shaking diameter of 2.5cm at 120rpm (New Brunswick S241i). Cells were transfected using PEIMax 40K (Polysciences) as a transfection reagent, at a PEI:DNA ratio of 3:1. Supernatants were harvested after 7 days and purified using *Gallanthus nivalis* lectin (Vector Labs) affinity chromatography, using an elution buffer of 1M Mannose (Sigma-Aldrich) in PBS. Any MGRM8 Env gp120 used for immunisation was tested for endotoxin contamination prior to use (Charles River).

#### Generation of murine FC mAbs

CD4bs bnAbs for infusion in this study were initially discovered in humans, and therefore required reformatting into a murine IgG format for infusion to the TIg mice. The murine IgG source vector for modification was a generous gift from Katie Doores (Kings College London). The native CH1 and hinge regions were excised and a gene string (GeneArt) encoding the human CH1 and hinge regions inserted via recombination-based assembly cloning (NEBuilder HiFi DNA Assembly kit, New England Biolabs), since these regions can modulate specificity of the variable region for its antigen.^44^ Gene strings encoding the V_H_ sequence of the bnAbs used in this study were then inserted by Gibson assembly to produce the murine FC (muFC) mAbs used in this study. These CD4bs mAbs were F105, PGV04, and NIH45-46.^23^^,^^45^^,^^46^ Plasmids encoding these mAbs were transfected into the HEK293F cells as described above, and transfection proceeded for 6 days, after which supernatant was purified using protein G Sepharose columns (GE Healthcare), using an elution buffer of 0.2M glycine at pH 2.2.

#### MuFC mAb binding by Env gp120 ELISA

To compare binding to parental (fully human) mAbs ELISA required immobilisation of MGRM8 Env gp120 to high binding 96 half-well plates (Corning), using 50μL of 1μg/mL protein, overnight at 4°C. Unbound Env gp120 was then removed by washing 3 times using 100μL 1X PBS (Gibco) with 0.05% Tween20 (PBS-T; Sigma-Aldrich), before blocking at room temperature using 5% milk powder (Marvel) in PBS for 2 hours. The blocked plates were then washed 3 times using 100μL of PBS-T. Titrations of either muFC or parental mAbs were created using 1:4 dilutions from an initial concentration of 10μg/mL, and 50μL per well was added in duplicate. This primary antibody was allowed to bind for 2 hours at room temperature before being removed by washing 3 times using 100μL PBS-T. The secondary antibody was then added at a 1:1000 dilution, at a volume of 50μL. For muFC mAbs, this was polyclonal AffiniPure Alkaline phosphatase (AP)-conjugated goat anti-mouse IgG (Jackson Immunoresearch, 115-055-071) and for parental human mAbs this was polyclonal AffiniPure AP-conjugated goat anti-human IgG (Jackson Immunoresearch, 109-055-098). These secondary mAbs were allowed to bind at room temperature for 1 hour, before plates were washed 6 times using 100μL PBS-T. Finally, AP substrate (Sigma-Aldrich; 5μg/mL) was added to the plate, and the plates read on a SpectraMax M5 plate reader (Molecular Devices), using an absorbance of 405nm. Colour was allowed to develop until the most concentrated sample reached an optical density of between 3.6 and 3.9.

#### Env gp120 muFC competition ELISA

Env gp120 immobilisation and blocking was performed as described above. Following this, a 1:4 dilution series of muFC mAbs were prepared, at a starting concentration of 25μg/mL, which was allowed to bind the immobilised Env gp120 for 2 hours at room temperature. Unbound mAb was removed by washing 3 times using 100μL of PBS-T. After this, 1μg/mL of the relevant parental mAb was added to the same well (i.e. muFC NIH45-46 primary antibody received fully human NIH45-46), to test for binding competition towards the same epitope. A non-epitope specific control was also tested in a separate well. The human mAbs were allowed to bind for 1 hour at room temperature, after which unbound antibody was removed and plates were washed 3 times using 100μL of PBS-T. Binding of parental mAb was then assessed by adding 50μL of a 1:1000 dilution of AffiniPure AP-conjugated goat anti-human IgG, and binding was allowed to proceed for 1 hour at room temperature. Removal of secondary antibodies and colourimetric detection proceeded as written previously.

#### Epitope analysis of fully human bnAbs

Structures 3U7Y and 3SE9 for NIH45-46 and PGV04 respectively were downloaded from the PDB^23^^,^^47^ and loaded into Chimera software v1.16 ^43^. In both structures, the Env protein was coloured grey, with the V_H_ and V_K_ chains of the antibodies coloured dark and light blue, respectively. The D368 residue in the gp120 was identified, and AAs within 4 Å were identified and coloured magenta. To compare the regions bound by each bnAb, their V_H_-gp120 contacts were downloaded manually extracted from PDBsum and processed using R.^48^^,^^49^

#### Immunisation of mice

Mice were maintained by staff in the *In vivo* Sciences and Delivery group in GSK. Immunisation groups comprised 10 mice. Each immunisation regimen was then divided into 2 groups of 5, who received the Env gp120 immunogen on sequential days, to allow more cells from each mouse to be sorted after each final harvest. Each mouse received a total of 5x 20μg Env gp120 immunisations spaced two weeks apart, and serum samples were taken for titre quantification 7 days following immunisations 2, 3 and 4. HIV Env gp120 was delivered subcutaneoulsy into both fore legs and hocks, with the exception of the final boost which was delivered intraperitoneally. The escalating affinity muFC HIV bnAbs were infused on days 17, 31, and 45, using 45μg per infusion per mAb as shown in Figure 1B. Tissues were harvested 3 days following the final immunisation.

#### Titre quantification by ELISA

Titre quantification by ELISA was performed by immobilising MGRM8 Env gp120 or MGRM8 D368R Env gp120 to a 96 well MaxiSorp treated high protein binding ELISA plates (Thermo Fisher), using 50μL per well at 1μg/mL. Immobilisation occurred at 4°C overnight. Excess antigen was removed by washing three times using 300μL PBS-T, prior to blocking using a BSA blocking buffer for 2 hours at room temperature with gentle agitation. Following removal of the blocking buffer and washing 3 times with 300μL PBS-T, titrations of murine serum samples were added (100μL per well). These began at a 1:1000 dilution using PBS-T, alternating between dilutions of 1:3 and 1:3.3, to a final dilution of 1:1000000 (7 dilutions per sample in total). Titrations were allowed to bind for 1 hour at room temperature, after which unbound serum was removed and plates were washed 3 times using PBS-T. A goat anti-mouse IgG Fc secondary antibody, conjugated to horseradish peroxidase (HRP) (Biorad; STAR120), was diluted 1:10000 using PBS, and 100μL added to each well. The secondary antibody was allowed to bind for 1 hour at room temperature, before unbound antibody was removed and plates were washed 6 times using 300μL of PBS-T. Colourimetric detection of bound serum was then performed by adding 100μL of room temperature 3,3′,5,5′-tetramethylbenzidine (TMB; Sigma-Aldrich), as a substrate for the HRP. This was incubated for 10 minutes at room temperature with gentle agitation, and the reaction stopped by addition of 100μL 1M H2SO4 (Sigma-Aldrich). Absorbance values were then read using a SpectraMax M5 plate reader (Molecular Devices), using a wavelength of 450nm. A dilution was deemed positive for Env gp120 binding if its absorbance exceeded the background threshold, defined as the mean of negative control wells (no serum added) multiplied by 3. A mouse was deemed appropriate for tissue harvesting and B cell isolation if its titre exceeded the background at the 1:100000 dilution. AUC analysis was carried out using the auc function of the MESS R package,^50^ and curves entirely below the baseline were assigned a value of 1. ABC analysis was carried out by subtracting the AUC calculated for the mutant D368R MGRM8 Env gp120 from the AUC calculated for the wild-type MGRM8 Env gp120.

#### Biotinylation of antigens for cell sorting

Both MGRM8 Env gp120 and BG505 D368R Env gp120 were used as probes for cell sorting therefore required biotinylation. By using different strains of Env gp120 as cell sorting probes CD4bs B cells were enriched while allowing more heterogeneity in the non-CD4bs specific cells, to assess any non-epitope specific effects of mAb treatment. Proteins were biotinylated using the EZ-Link™ Sulfo-NHS-LC-Biotin kit (Thermo Fisher), using manufacturers recommendations. Following this, excess unbound biotin was removed by repeated centrifugation and filtration through Amicon 30kDa centrifugal concentrators (Merck-Millipore). Degree of labelling (DoL) was then quantified using the QuantTag Biotin Quantitation kit (2B Scientific), and proteins were deemed appropriate for cell sorting if the DoL was approximately 3 biotins per protein. These were stored at -80°C until the day of cell sorting. On the day of sorting, the biotinylated MGRM8 Env gp120 was complexed with streptavidin conjugated phycoerythrin and the biotinylated BG505 D368R Env gp120 was complexed with streptavidin conjugated allophycocyanin (APC) for 10 minutes at room temperature (in the dark), prior to cell labelling.

#### Cell sorting of Env gp120-specific B cells

Following the immunisation regimen, bone marrow, SPL and LN tissues were harvested into EX-CELL 610-Human Serum Free medium (Sigma-Aldrich), with separate mice being retained separately. LN and spleen were used for selection of Env gp120-specific B cells, using established methods. Briefly, tissues were homogenised through a 40μm cell strainer (Corning), and spleen and bone marrow tissues underwent red blood cell (RBC) lysis using the Hybri-Max RBC lysis reagent (Sigma-Aldrich).

For cell sorting, samples were pooled equally between mice in the same immunisation group, producing a mixed pool of 5x10^7^ cells derived from different mice. The antigen-fluorochrome tetramers produced previously were then added to the cell pool, which was incubated on ice in the dark for an hour. B cells were then incubated with murine Fc block (Biolegend), after which the immunophenotyping cocktail of labelled antibodies was added. This contained Ter119 PerCP-Cy5.5 (BD Biosciences), CD3 PerCP-Cy5.5 (Biolegend), Ly6G PerCP-Cy5.5 (BD Biosciences), CD11c PerCP-Cy5.5 (BD Biosciences), B220 PE-Cy7 (Biolegend), IgG BV421 (Biolegend) and IgM BV510 (Biolegend). Cells were stained with this cocktail for 30 minutes on ice and in the dark, before pelleting and reconstituting in 1mL of PBS containing 2% FBS and 1mM ethylenediaminetetraacetic acid, with a 1:1000 dilution of Near-IR Dead Cell Stain (Thermo Fisher). Cell sorting proceeded using a BD FACSAria Fusion Cell Sorter (BD Biosciences). Cells were gated to exclude those that were positive for Ter119, CD3, CD11c, Ly6G, IgM or BG505 D368R Env gp120. The cells selected were therefore positive for B220, IgG and MGRM8 Env gp120, without the aforementioned depletion markers.

The selected cells were deposited into individual wells of a semi-skirted 96 well PCR plate (Applied Biosystems), containing reverse transcription (RT) mastermix. This mastermix contained random hexamers (Genscript), as well as RNaseOUT (Thermo Fisher), SuperScript III™ (Thermo Fisher) and associated buffers. Samples then underwent RT, using the following conditions; 42°C for 10 minutes; 25°C for 10 minutes; 50°C for 60 minutes and 94°C for 5 minutes, before final store conditions at 4°C. Post-sorting analysis was performed using FlowJo.

#### Magnetic bead selection of plasma cells

The bone marrow tissues that underwent RBC lysis were then depleted for non-PCs using magnetic bead depletion. PCs were incubated with biotinylated antibodies targeting Ter119 (StemCell), CD11b (Biolegend), CD71 (Biolegend), CD105 (Biolegend), CD3 (Biolegend), CD11c (eBioscience), CD34 (Biolegend), CD5 (Biolegend), IgD (Southern Biotech), CD49 (Biolegend) and IgM (Biolegend). EasySep Streptavidin RapidSpheres (StemCell) were then added and samples were separated on a magnetic tube stand. The unbound PCs in the supernatant were then aspirated and cryopreserved for downstream Next Generation Sequencing (NGS). Unlike samples for single cell sorting, the bone marrow tissues from different mice were retained separately.

#### Single cell PCR of Env gp120-specific mAbs

cDNA produced previously underwent two rounds of nested PCR, designed to amplify paired V_H_ and V_K_ sequences for eventual mAb transfection. Forward primers annealed in the leader region and reverse primers annealed in the constant region in both reactions. To express the mAbs, transcriptionally active PCR (TAP) fragments were produced using established methods.^51^ The V_H_ and V_K_ TAP fragments were submitted to GeneWiz for low throughput sanger sequencing. Transfection was carried out in Expi293F cells, using the ExpiFectamine Transfection kit (Gibco). Cognate V_H_ and V_K_ sequences were assembled into a sterile U-bottom 96 well plate (Thermo Fisher), using 0.35μg of each chain, after which they were combined with a 1:20 dilution in OptiMEM of Expifectamine transfection reagent (Gibco). The transfection complexes were allowed to form by incubating at room temperature for 20 minutes, after which they were added to Expi293F 2.0x10^6^ cells in sterile U bottom deep well plates (Corning), and cultured for 18 hours at 37°C, 8% CO_2_ at 950rpm with an orbital shaking diameter of 5mm. Next, transfection enhancers were added and cells allowed to express the mAbs for a further 4 days before supernatant harvest.

#### mAb binding quantification by SPR

Harvested mAb supernatant samples were analysed for binding using the Carterra LSA (Carterra Bio), without additional purification. Briefly, mAb containing supernatant samples were diluted 1:4 using Expi293F media and 200μL of this dilution was reformatted into 384 deep well plates (Brooks). This was sealed with pre-pierced 384 deep well plate seals and loaded into the Carterra LSA. This supplied enough volume to characterise mAb binding to wild-type MGRM8 Env gp120 or the D368R mutant. MGRM8 Env gp120 and MGRM8 D368R Env gp120 were each serially diluted from 1μM to 0.24nM using HBS-EP buffer (Cytivia) and 300μL per dilution was dispensed into a round bottom 96 well plate, which was also loaded into the Carterra. Assay setup involved initial capture of mAbs on a protein A/G Carterra LSA Sensor Chip (Carterra). SPR was performed at 30°C, with the following stages; 5 minutes to capture mAb to the chip; 1 minute of baseline acquisition; 5 minutes of antigen association; and finally 10 minutes of dissociation. Each of these cycles was followed by 2 lots of 30 second regenerations using NaOH (Sigma-Aldrich).

After data acquisition, the sensorgrams were then curated to ensure faithful representation of the experimental data was obtained, defined as having sensorgram residual values of less than 10. Sensorgrams were also manually curated to confirm the results. Only mAbs whose sensorgrams met the previous criteria had their K_D_ values analysed, but wells with demonstrable binding to either antigen were considered as binders for annotation of the mAb sequences, regardless of the quality of their sensorgrams. Epitope specificity was assigned based on this binding data. mAbs with binding to the wild-type MGRM8 Env gp120 but no binding to the MGRM8 D368R Env gp120 were assigned as CD4bs, with knockout status. mAbs with binding to both antigens, but with at least 10-fold weaker binding to the D368R Env gp120 were assigned as CD4bs, with knock-down status since their binding was reduced but not ablated by the mutation. mAbs with binding to MGRM8 D368R Env gp120 within 10-fold of their binding to wild-type MGRM8 Env gp120 were assigned as non-CD4bs-specific.

#### Bulk NGS of enriched plasma cell samples

For bulk NGS of IgG PCs, the cryopreserved PCs first underwent RNA extraction using the RNeasy Mini Kit (QIAGEN). From the purified RNA, 5μg per sample was selected for NGS library generation in triplicate, and its volume adjusted to 15μL per reaction. Library generation proceeded similarly to previously described.^52^ Briefly, mRNA was incubated with a primer annealing mastermix containing a 13bp UMI tagged IgG specific primer, dNTPs (Thermo Fisher) and molecular biology grade water (Sigma-Aldrich), to a final volume (including the RNA sample) of 17.75μL. The IgG primer also contained the Illumina read 2 site at its 5’ terminus to minimise the size of the amplicon. Primer annealing proceeded at 65°C for 5 minutes, followed by incubation on ice for 5 minutes. This mixture was then supplemented with an RT mix containing SuperScript IV™ reverse transcriptase (Thermo Fisher; 100 units), RNaseOUT™ (Thermo Fisher; 2mM), dithiothreitol (DTT) (Thermo Fisher; 5mM) and SuperScript™ First Strand (Thermo Fisher). Concentrations provided reflect the concentration in the fully assembled RT reaction. RT proceeded at 50°C for 45 minutes before enzyme inactivation at 80°C for 10 minutes, as published previously.^31^ Any residual RNA:cDNA hybrids were then treated with 2 units of RNase H (Thermo Fisher). The result is a cDNA library whose constituent molecules all contained a UMI to facilitate bioinformatic error correction.

cDNA was purified using 1.0X AMPure XP magnetic beads (Beckman Coulter), and purified DNA was subsequently eluted in 40μL of molecular biology grade water (Sigma-Aldrich). Library generation then continued by multiplex PCR, using a mix of leader-specific forward primers, previously validated within GSK to capture all individual V_H_ genes evenly. These anneal to the leader sequence in the V_H_ cDNA and also introduce the Illumina read 1 site on their 5’ terminus. The reverse primer anneals to the Illumina read 2 site introduced by the RT primer. This NGS PCR1 mastermix is formulated with Q5 Hot-Start High Fidelity Mastermix (NEB), leader-specific forward primer mix (100nM per primer), and the reverse primer (100nM), in a volume of 30μL. The choice of a proof-reading, high-fidelity polymerase was made to reduce errors during the PCRs. This was supplemented with 20μL of purified cDNA. The reaction conditions for the NGS PCR1 were as follows; denaturation at 98°C for 50 seconds; 11 cycles of touchdown PCR comprising denaturation at 98°C for 10 seconds, primer annealing at 68°C to 63°C (0.5°C temperature decrease per cycle) for 20 seconds, and extension at 72°C for 20 seconds; final extension at 72°C for 120 seconds. The limited PCR cycling was favoured to reduce biasing the amplification to more prevalent V_H_ sequences.

This NGS PCR1 product was then purified using 0.6X AMPure XP magnetic beads (Beckman Coulter), and purified DNA was subsequently eluted in 22μL of molecular biology grade water (Sigma-Aldrich). The NGS PCR2 uses a common forward primer that anneals to the read 1 site introduced in NGS PCR1 and completes the Illumina P5 adapter. The reverse primer again recognises the Illumina read 2 site, contains a sample specific barcode to facilitate library multiplexing, and completes the Illumina P7 adapter. The NGS PCR2 mastermix is formulated identically to the NGS PCR1 mastermix, except using the aforementioned primers. It was supplemented with 20μL of purified NGS PCR1 DNA, and the NGS PCR2 reaction proceeded thus; denaturation at 98°C for 50 seconds; 8 cycles of touchdown PCR comprising denaturation at 98°C for 10 seconds, primer annealing at 72°C to 68.5°C (0.5°C temperature decrease per cycle) for 20 seconds, and extension at 72°C for 20 seconds; 17 cycles of standard PCR comprising denaturation at 98°C for 10 minutes, primer annealing at 68°C for 20 seconds, and extension at 72°C for 20 seconds; final extension at 72°C for 120 seconds.

NGS PCR2 product was then purified using 0.6X AMPure XP magnetic beads (Beckman Coulter). These libraries were then quantified for size and concentration using the TapeStation 4150 (Agilent Technologies) and QIAcuity Digital PCR System (QIAGEN).

#### Loading the samples to the Illumina MiSeq

The libraries were sequenced using a MiSeq (Illumina) using the MiSeq reagent kit v3. The module used during sequencing was ‘GenerateFASTQ – 3.0.1’, with default chemistry. The read lengths were 325bp and 287bp for reads 1 and 2 respectively, with an 8bp sample indexing read, to maximise the higher quality forward read.^53^ Libraries were diluted to 10nM and a maximum of 10 were pooled for each sequencing run before loading to the MiSeq, at a final concentration of 10pM containing a 10% spike in of PhiX control library (Illumina).

#### Processing of sanger sequencing samples

Ab1 files of low throughput sequencing samples were processed using the sangerseqR R package.^54^ This produced a fasta file from the ab1 files received from GeneWiz, which was later concatenated with the fasta files output following NGS sequence processing.

#### Processing of NGS samples

The fastq files obtained following NGS were processed using the pRESTO package from the Immcantation suite of tools, based on the ‘UMI Barcoded Illumina MiSeq 2x250 BCR mRNA’ example workflow.^55^ Briefly, sequences were filtered for quality (phred score ≥ 20) and primer regions masked. UMIs were then copied across each pair of reads, and sequence alignment performed within each read group using muscle. A consensus sequence was then built for each read per UMI group, and full-length sequences constructed. Duplicate sequences were then collapsed within each UMI group, and sequences with only a single read were discarded. This stage allowed errors within the read group to be corrected. The fasta file output from this process was joined with the V_H_ fasta file derived from low throughput sequencing for sequence annotation and lineage clustering.

#### Ig sequence annotation and clustering

The fasta file produced above was annotated using IgBLAST, accessed using Change-O from the Immcantation framework.^56^^,^^57^ Change-O was used to perform Ig gene assignment on the sequences, as well as other characteristics such as identifying CDR and FWR regions. Sequences were coarsely clustered into groups based on having identical IGHV and IGHJ gene assignments as well as identical CDR3 lengths. These were then clustered into lineages based on 90% CDR3 amino acid identity from the normalised distance matrix, using single-linkage hierarchical clustering. Clustering both low and high throughput sequences together links the B cells in peripheral tissues with PCs in the bone marrow. These clusters were then used to infer a single germline sequence per cluster.

#### Bioinformatic analysis of V_H_ sequences

The processed sequences were analysed using R v4.1.2, using the tidyverse suite of packages for most data processing.^49^^,^^58^ B cell networks were constructed using igraph,^59^ and amino acid mutations were counted using the observedMutations function from the shazam R package.^60^ Gini index was calculated using the gini function from the DescTools R package.^61^ Phylogenetic trees of B cell lineages were estimated using IgPhyML, via the BuildTrees.py script in Change-O.^57^^,^^62^^,^^63^ All lineages from low throughput sequencing data were analysed, providing they had 5 or more members, whereas only selected lineages were analysed for high throughput sequencing datasets owing to computational intensity. The CDR3 was excluded from analysis as recommended by the authors, to prevent SHM biases in this region affecting selection measurements. The 95% confidence interval for the ω (dN/dS; nonsynonymous to synonymous mutation ratio) was computed for both FWR and CDRs. The total cophenetic index (TCI) was calculated using the TotalCopheneticIndex function from the TreeTools package.^64^^,^^65^ The root to tip distance was calculated using the distRoot function from the adephylo package.^66^

### Quantification and statistical analysis

Statistical analysis was also performed in R v4.1.2. Data was assessed for a Gaussian distribution using the shapiro.test function. A T test (t.test function) was used if data was Gaussian, and a Mann-Whitney U test (wilcox.test function) if it was not. All statistical functions were from the stats R package.^49^ Data was visualised using the ggplot2 package from the tidyverse suite of packages, and the associated ggtree package for visualising phylogenetic trees.^58^^,^^67^

## Supplemental information

Document S1. Figures S1–S4

Table S1. Table illustrating the frequency of lineages recovered from Sanger sequencing of antigen-specific B cells in individual mice from NGS of bone marrow resident IgG plasma cells, related to Figure 2 Table provides B cell lineage IDs on the left side (rows), and their frequency in individual mice per immunization regimen (columns). Highlighted rows illustrate the high affinity lineages identified in Figure 2E. The number of mice that possessed each lineage was counted, which was used to calculate a public score (number of mice with the lineage divided by the total number of mice; columns S and T). Size bins (column U) were assigned from 1 (lineage is present in one mouse) to 17 (lineage is present in all mice), and the number of lineages meeting this criteria counted (column V).

Table S2. Characteristics of B cell lineages discovered by Sanger sequencing, related to Figure 3 Table summarizing the SHM and phylogenetic characteristics of the lineages discovered by Sanger sequencing. Convergent lineages discovered in both immunization regimens are colored orange, and lineages that had their phylogenetic trees displayed in Figure 5 are marked within boxes. Lineages with fewer than 5 members total (across both immunization groups) are not displayed, since phylogenetic analysis was not carried out.
