# Supplementary material for: High affinity mAb infusion can enhance maximum affinity maturation during HIV Env immunization
Source: iScience. 2024 Mar 11;27(4):109495. doi: 10.1016/j.isci.2024.109495 (PMC10973984; doi:10.1016/j.isci.2024.109495)
Supplement: Document S1. Figures S1–S4 [file mmc1.pdf]

iScience, Volume *27*

Supplemental information

High affinity mAb infusion can enhance maximum affinity maturation during HIV Env immunization

Peter Thomas, Chloe Rees-Spear, Sarah Griffith, Luke Muir, Emma Touizer, Raiees Andrabi, Richard Priest, Jennifer Percival-Alwyn, Darryl Hayward, Amanda Buxton, William Traylen, Benny Chain, Trevor Wattam, Irene Sanjuan Nandin, and Laura E. McCoy

Fig. S1. Anti-Env gp120 titre comparison between immunisation strategies, related to Figure. 1

Characterisation of the anti-Env serum response within separate immunisation regimens, by MGRM8 Env gp120-specific ELISA. **(A)** All ELISA curves from the serum samples presented in Fig. S2B and Fig. S2C. Plot columns reflect the serum sample and plot rows the immunisation regimen. The top two panels reflect binding to wild-type MGRM8 Env gp120 and the bottom two panels reflect binding to MGRM8 D368R Env gp120. **(B)** Distribution of the logged (base = 2) area under the curve (AUC) values from ELISA, as shown in C for an individual mouse, at each bleed timepoint. The top panel reflects the AUCs for the wild-type MGRM8 Env gp120 and the bottom panel the AUCs for the D368R CD4bs Knockout MGRM8 Env gp120. Boxplots are separated by immunisation group. Pairwise Mann-Whitney tests were carried out, however no statistically significant differences ($p > 0.05$) were present between the groups at any timepoint. **(C)** Distribution of the logged (base = 2) area between the curve (ABC) values from ELISA, whereby the AUC for the CD4bs knockout Env gp120 has been subtracted from the AUC for the wild-type Env gp120. Pairwise Mann-Whitney U tests were carried out between each immunisation group per serum sample (*** $p \leq 0.001$, ** $p \leq 0.01$, * $p < 0.05$).

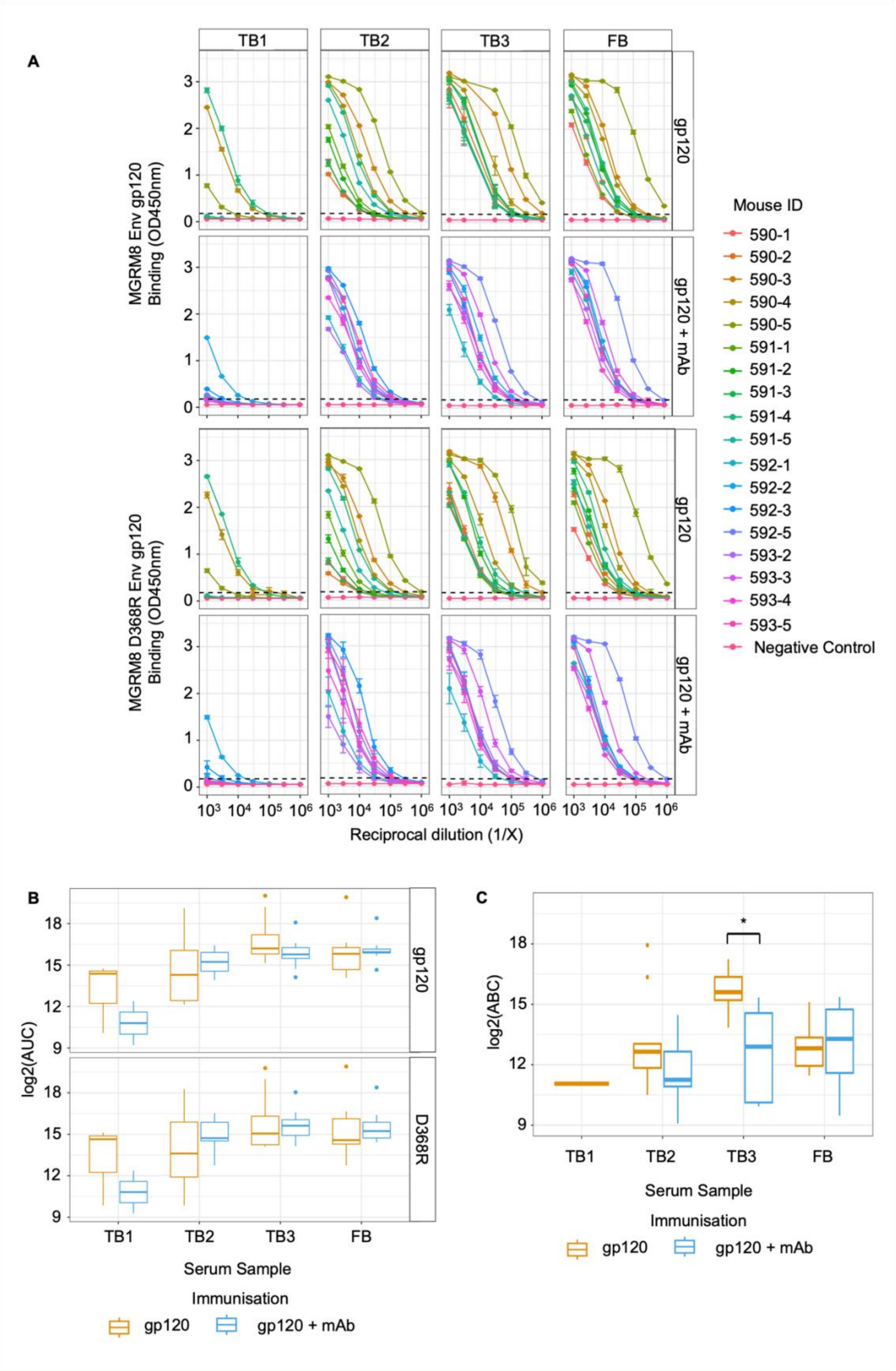
A
TB1
TB2
TB3
FB
gp120
gp120 + mAb
MGRM8 Env gp120 Binding (OD450nm)
MGRM8 D368R Env gp120 Binding (OD450nm)
Reciprocal dilution (1/X)
Mouse ID
590-1
590-2
590-3
590-4
590-5
591-1
591-2
591-3
591-4
591-5
592-1
592-2
592-3
592-5
593-2
593-3
593-4
593-5
Negative Control
B
log2(AUC)
gp120
D368R
Serum Sample
Immunisation
gp120
gp120 + mAb
C
log2(ABC)
*
TB1
TB2
TB3
FB
Serum Sample
Immunisation
gp120
gp120 + mAb

Fig. S2. Isolation of Env gp120-specific B cells by single cell sorting, related to Figure. 1

Following the immunisation regimen, CD4bs-specific cells were enriched using single cell sorting for mAb expression and sequence analysis. **(A)** Representative flow cytometry plots demonstrating Env-gp120 binders ($B220^+/IgM^-/IgG^+$) that were sorted for further analysis based on binding of MGRM8 gp120-PE and BG505 D368R gp120 APC for the control immunisation group (top) and mAb infusion immunisation group (bottom). **(B)** Bar charts depicting frequency of IgM (left) and IgG (right) positive events within the Live/Lymphocyte/B220+ B cell population.

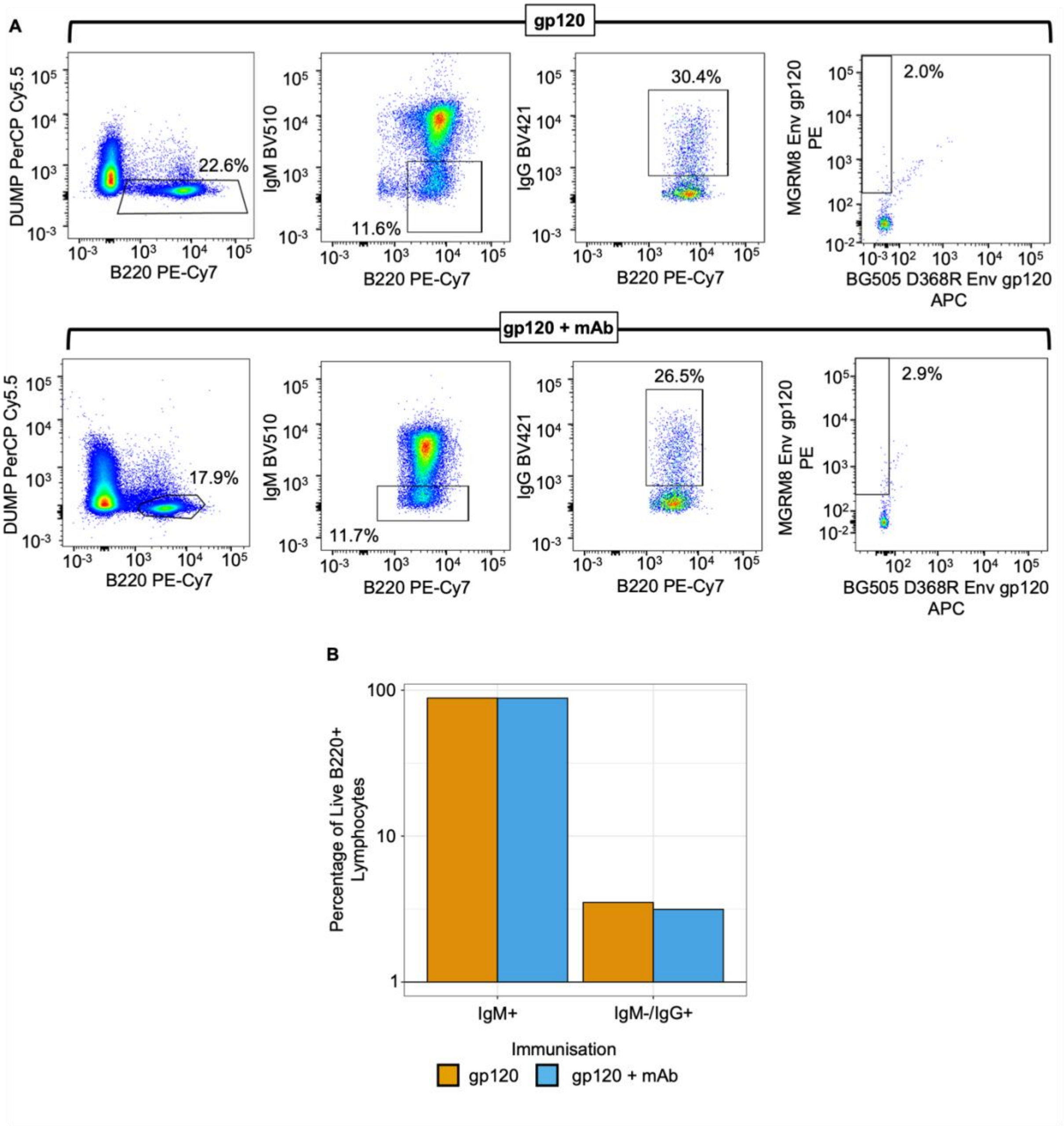
A
gp120
DUMP PerCP Cy5.5
B220 PE-Cy7
22.6%
IgM BV510
11.6%
IgG BV421
30.4%
MGRM8 Env gp120 PE
BG505 D368R Env gp120 APC
2.0%
gp120 + mAb
17.9%
11.7%
26.5%
2.9%
B
Percentage of Live B220+ Lymphocytes
IgM+
IgM-/IgG+
Immunisation
gp120
gp120 + mAb

Fig. S3. Binding footprint of the two IGHV1-2 bnAbs used for infusion, related to Figure. 1

Structural analysis of two D368 engaging CD4bs specific bnAbs, to confirm that the selected mAbs for infusion would compete with B cells isolated by single cell sorting. **(A)** Summary of gp120 residues bound by two mAbs used for infusion, using structures for NIH45-46 (left) and PGV04 (right) from PDB structures (3U7Y & 3SE9 respectively). The x axis reflects mAb AA residues binding the Env gp120, and the y axis reflects the residues bound on the Env gp120. Molecular contacts for each structure were manually curated from PDBsum. Residues within 4Å of each other (between gp120 and mAb structures) that did not form bonds are not displayed. Colour of the squares reflects if either mAb forms hydrogen bonds with the gp120 at the specific AA residue. Black spots reflect if a salt bridge is formed. The D368 residue in the Env gp120 and mAb AA residues in direct contact with it are coloured blue. **(B)** Structures depicting engagement of gp120 D368 residue by NIH45-46 (left; PDB structure 3U7Y) and PGV04 (right; PDB structure 3SE9). Hydrogen bonds formed between mAb V_H chain and D368 are shown in magenta. Images generated using Chimera.

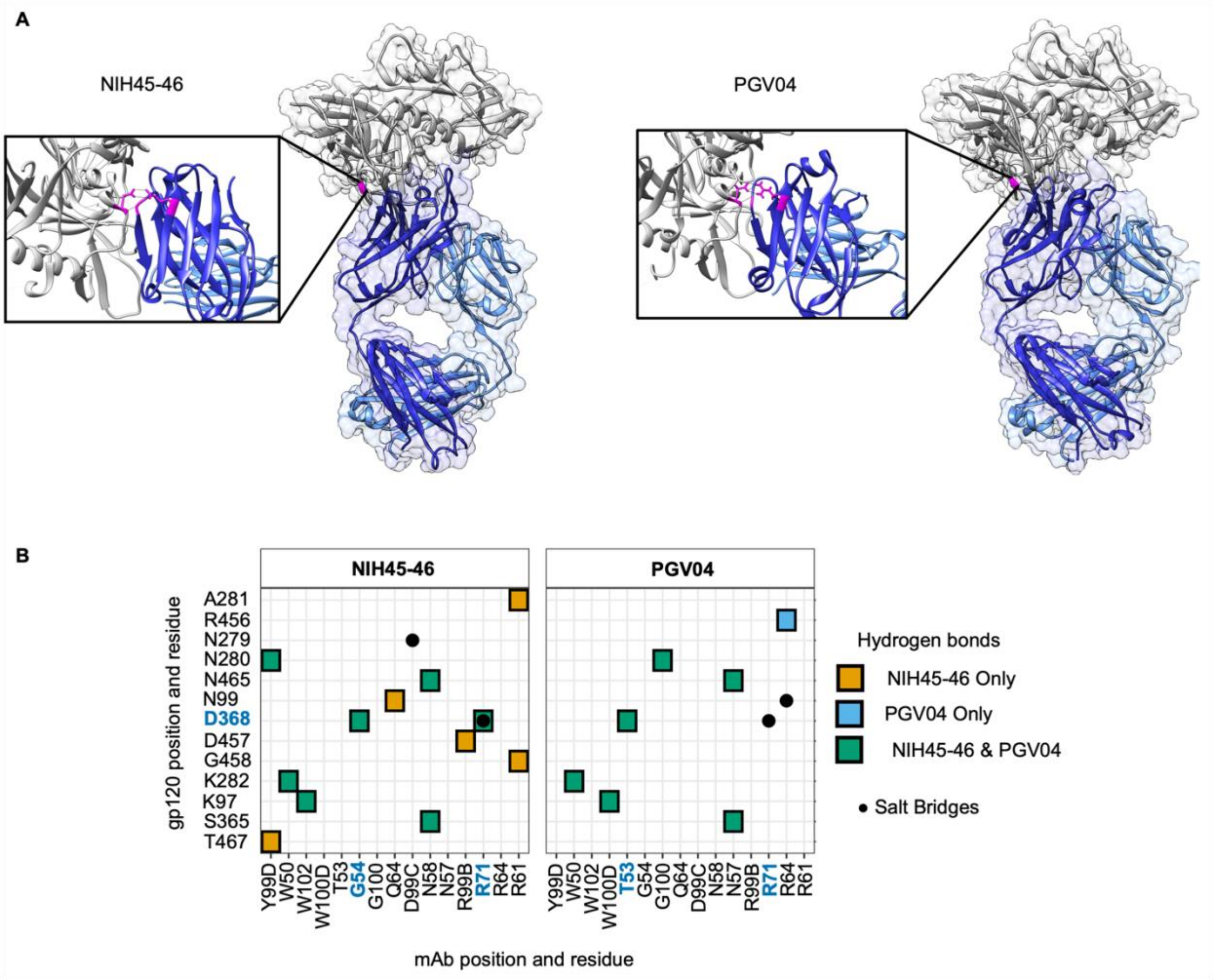
A
NIH45-46
PGV04
B
NIH45-46
PGV04
gp120 position and residue
A281
R456
N279
N280
N465
N99
D368
D457
G458
K282
K97
S365
T467
Y99D
W50
W102
W100D
T53
G54
G100
Q64
D99C
N58
N57
R99B
R71
R64
R61
mAb position and residue
Hydrogen bonds
NIH45-46 Only
PGV04 Only
NIH45-46 & PGV04
Salt Bridges

Fig. S4. Gini index of clonal lineages and V_HJ_H gene recombinations, related to Figure. 2

The Gini index was calculated using the R package DescTools to describe the size distributions within clonal lineages (left) and V_HJ_H gene recombinations (right) for Sanger sequencing data. A value of 0 reflects perfect equality, whereas a value of 1 reflects perfect inequality. Inequality was assessed via repeated sampling (n = 20) of 25 or 50 sequences per group for CD4bs and Non-CD4bs V_H sequences respectively (different values to account for the different group sizes). Data was Gaussian therefore significance was assessed by ANOVA and with Tukey's Honest Significant Difference test (*** $p \leq 0.001$, ** $p \leq 0.01$, * $p < 0.05$).

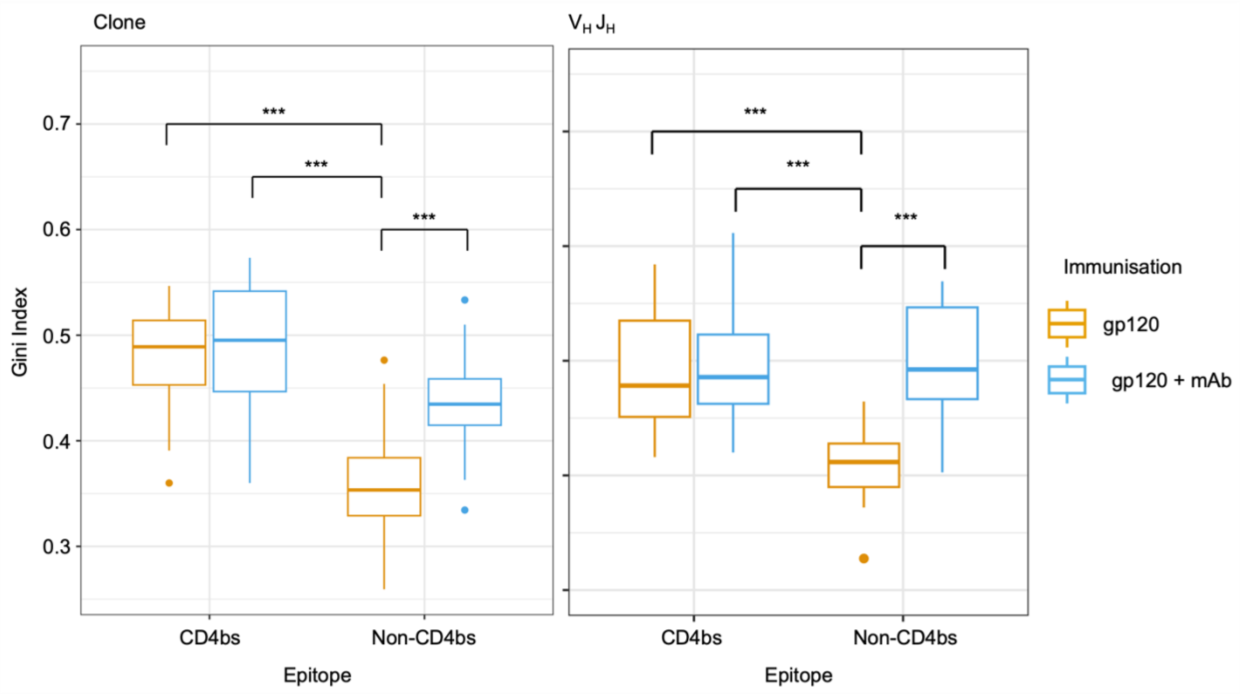
Clone
$V_H J_H$
0.7
0.6
0.5
0.4
0.3
Gini Index
***
***
***
***
***
***
CD4bs
Non-CD4bs
Epitope
CD4bs
Non-CD4bs
Epitope
Immunisation
gp120
gp120 + mAb
